# A Closed-Loop Optogenetic Platform

**DOI:** 10.3389/fnins.2021.718311

**Published:** 2021-09-10

**Authors:** Dimitrios Firfilionis, Frances Hutchings, Reza Tamadoni, Darren Walsh, Mark Turnbull, Enrique Escobedo-Cousin, Richard G. Bailey, Johannes Gausden, Aaliyah Patel, Dorian Haci, Yan Liu, Fiona E. N. LeBeau, Andrew Trevelyan, Timothy G. Constandinou, Anthony O'Neill, Marcus Kaiser, Patrick Degenaar, Andrew Jackson

**Affiliations:** ^1^Neuroprosthesis Lab, School of Engineering, Newcastle University, Newcastle upon Tyne, United Kingdom; ^2^Digital Institute, Newcastle University, Newcastle upon Tyne, United Kingdom; ^3^Biosciences Institute, Faculty of Medical Sciences, Newcastle University, Newcastle upon Tyne, United Kingdom; ^4^Emerging Technologies and Materials Group, School of Engineering, Newcastle University, Newcastle upon Tyne, United Kingdom; ^5^Department of Electrical and Electronic Engineering, Imperial College London, London, United Kingdom; ^6^Department of Micro-Nano Electronics, Shanghai Jiaotong University, Shanghai, China; ^7^Care Research and Technology Centre, UK Dementia Research Institute, London, United Kingdom; ^8^School of Computing, Newcastle University, Newcastle upon Tyne, United Kingdom; ^9^School of Medicine, University of Nottingham, Nottingham, United Kingdom; ^10^Rui Jin Hospital, Shanghai Jiao Tong University, Shanghai, China

**Keywords:** optogenetics, closed-loop, neuromodulation, electrophysiology, open-source

## Abstract

Neuromodulation is an established treatment for numerous neurological conditions, but to expand the therapeutic scope there is a need to improve the spatial, temporal and cell-type specificity of stimulation. Optogenetics is a promising area of current research, enabling optical stimulation of genetically-defined cell types without interfering with concurrent electrical recording for closed-loop control of neural activity. We are developing an open-source system to provide a platform for closed-loop optogenetic neuromodulation, incorporating custom integrated circuitry for recording and stimulation, real-time closed-loop algorithms running on a microcontroller and experimental control via a PC interface. We include commercial components to validate performance, with the ultimate aim of translating this approach to humans. In the meantime our system is flexible and expandable for use in a variety of preclinical neuroscientific applications. The platform consists of a Controlling Abnormal Network Dynamics using Optogenetics (CANDO) Control System (CS) that interfaces with up to four CANDO headstages responsible for electrical recording and optical stimulation through custom CANDO LED optrodes. Control of the hardware, inbuilt algorithms and data acquisition is enabled via the CANDO GUI (Graphical User Interface). Here we describe the design and implementation of this system, and demonstrate how it can be used to modulate neuronal oscillations *in vitro* and *in vivo*.

## Introduction

Neuromodulation is an established therapy for many neurological conditions, and has been clinically approved in certain forms for epilepsy (Fisher et al., 2005) and Parkinson's disease (Raza et al., 2019), providing a means to control abnormal oscillatory dynamics in neural circuits. There are ongoing efforts to incorporate closed-loop control of stimulation based on real-time activity of local brain networks (Wang et al., 2015; Bouthour et al., 2019; Cagnan et al., 2019), for example using local field potentials (LFPs) (Buzsaki et al., 2012). The combination of electrophysiological recording and electrical stimulation have enabled new neuromodulation strategies (Jackson et al., 2006; Kokkinos et al., 2019; Lozano et al., 2019), but these are hampered by the recording artefacts associated with delivering electrical current to the tissue. Optogenetics (Deisseroth, 2011) provides a potential solution to this problem since light can be used to activate (or suppress) neurons without concurrent electrical artefacts, and moreover, the use of promoter-dependent expression of opsins allows stimulation to be targeted to specific neuronal sub-types. While there are many challenges to translating optogenetics to the human brain (Shen et al., 2020) including demonstrating the long-term safety and efficacy of opsin expression, an optogenetic therapy for the retina has recently been tested for the first time in humans (Sahel et al., 2021).

Several systems (Pashaie et al., 2015; Frank et al., 2019; Mickle et al., 2019; Zhou et al., 2019; Gagnon-Turcotte et al., 2020; Liu et al., 2020; Vazquez-Guardado et al., 2020; Yang et al., 2021) have been developed that utilise recording and stimulation techniques in order to support ongoing neuromodulation research. Systems such as the Open Ephys (Siegle et al., 2017), the BCI2000 (Schalk et al., 2004), the CLoSES (Zelmann et al., 2020) and the NeuroRighter (Newman et al., 2013), provide good paradigms of ongoing development, ranging from open-source Graphical User Interface (GUI) software to open-loop and close-loop electrophysiology. Moreover, there are systems that focus more on acquiring recordings from a large number of channels (Kinney et al., 2015; Putzeys et al., 2019; Steinmetz et al., 2021) and others, such as the Medtronic Inc. Summit RC+S (Bourget et al., 2015), that implement closed-loop neuromodulation within implantable systems suitable for human use.

**Figure 1:**
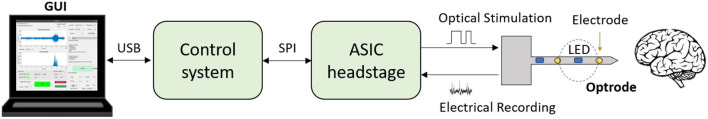
Schematic of a closed-loop optogenetic platform for neuromodulation research. Brain signals are recorded via electrodes and optical stimulation is delivered via micro-LEDs located on the shaft of an optrode. An Application Specific Integrated Circuit (ASIC) headstage implements recording and LED driving, and communicates with a microcontroller-based control system via an Serial Peripheral Interface (SPI). The control system receives the recorded data and processes it in real-time to deliver appropriate stimulation commands. The closed-loop algorithm is configured and monitored by the user via a Graphical User Interface (GUI). The GUI also allows for the acquired data to be stored for post-processing.

The system we propose here is an open-source^1^ closed-loop platform (Controlling Abnormal Network Dynamics using Optogenetics Control System—CANDO-CS)—shown in Figure 1—that enables optogenetic neural modulation based on custom hardware and algorithms. The system is being developed as part of the on-going progress in the CANDO Project^2^, aiming toward translation to a miniaturised implantable device for humans. To facilitate this, low-power electrical recording and optical stimulation are implemented by a CANDO3 Application Specific Integrated Circuit (ASIC), which is an improved version of the front-end electronics we have described previously (Ramezani et al., 2018). To ensure precise timing of real-time closed-loop algorithms, we use a microcontroller-based control system to interface with the front-end. Parameters are uploaded and data is acquired via a CANDO GUI running on a PC. To evaluate the efficacy of the custom hardware and algorithms, as well as to integrate with laboratory hardware, the system has been designed to be compatible with commercial components (e.g., Intan Recording controller, Intan headstage, NeuroNexus electrodes etc.). This enables verification of headstage and optrode configurations prior to miniaturisation and integration into an implantable system. Moreover, the versatility provided by the integration with commercial components means that our system is not limited to applications involving the custom ASIC and optrodes, but can also be used for a variety of closed-loop neuroscience experiments in a range of *in vitro* and *in vivo* laboratory scenarios.

## System Overview

**Figure 2:**
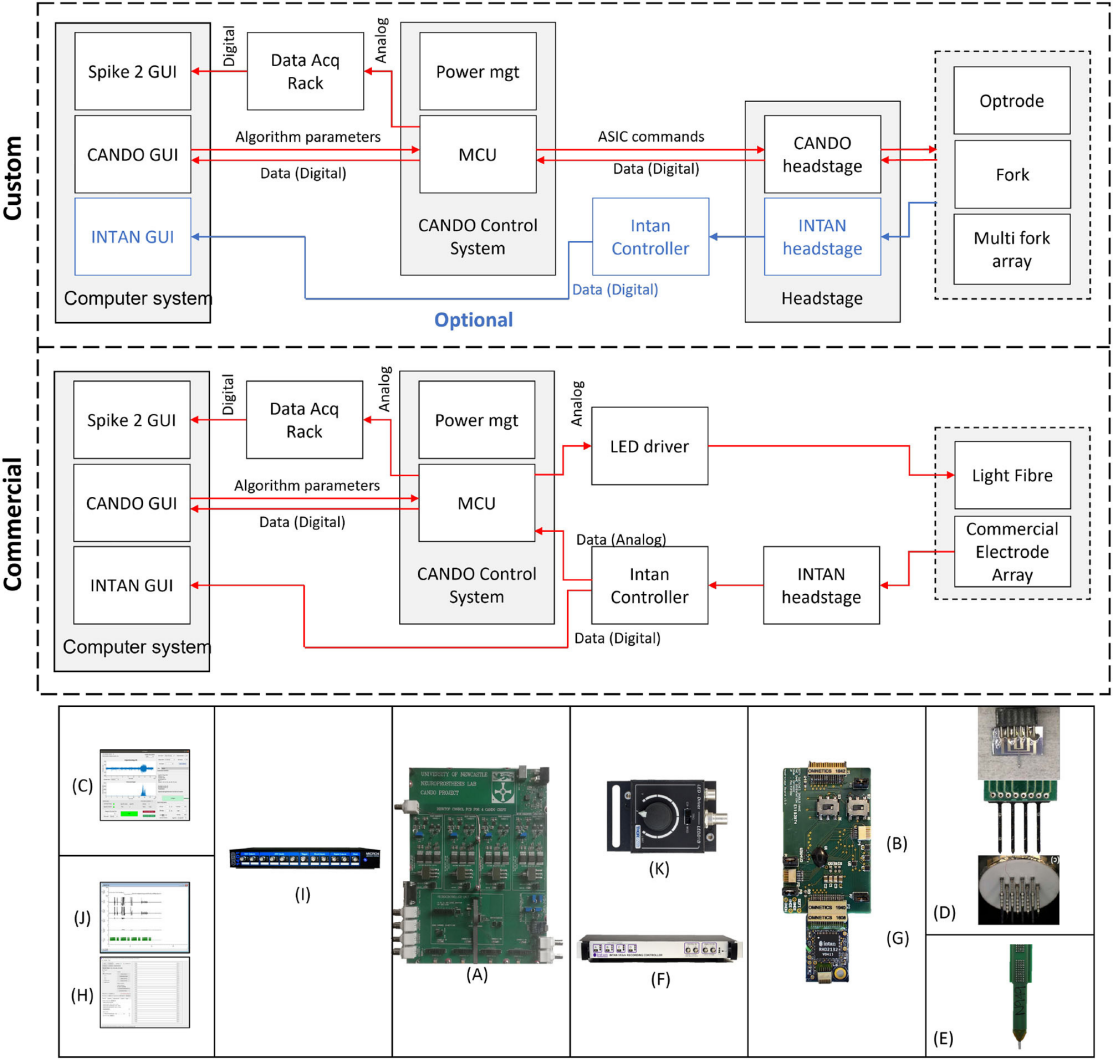
Overview of the full system. We show two configurations to illustrate how the system can be used in combination with custom components (*top*) as well as only commercial components (*middle*). Custom components include **(A)** CANDO Control System, **(B)** CANDO headstage, **(C)** CANDO GUI, and **(D)** CANDO Optrodes. Commercial components include **(E)** Commercial Electrode Array & Light Fibre, **(F)** Intan Recording Controller, **(G)** Intan headstage, **(H)** Intan GUI, **(I)** 1401-4 Data Acquisition System, **(J)** Spike2 GUI, and **(K)** T-Cube LED Driver.

An overview of the system components is provided in Figure 2. We show two possible configurations to illustrate (*top*) how the system can be used in combination with other custom CANDO components, and (*middle*) how the system can be used with commercial components. The parts that have been developed in-house are the CANDO-CS (Figure 2A), the CANDO headstage (Figure 2B), the CANDO Graphical User Interface (GUI) (Figure 2C) and the CANDO optrodes (optrode, fork, and multi-fork array) (Figure 2D). The commercial components include a commercial electrode array (Figure 2E), an RHD recording controller (Figure 2F) for data acquisition, an RHD 32-channel recording headstage (Figure 2G) for electrical recording from Intan Technologies, and a Micro1401-4 Data Acquisition System (Figure 2I) from CED. Intan technologies and CED have their own data acquisition software, namely RHX (Figure 2H) and Spike2 (Figure 2J), respectively. The CANDO-CS is controlled via the CANDO GUI, where the user can configure recording and stimulation hardware, stimulation protocols and algorithm parameters. The 36-way Omnetics connector on the CANDO headstage connects to a range of standard recording probes (e.g., NeuroNexus arrays). Moreover, stimulation through optic fibres can be controlled via connection to commercial LED-drivers, such as the T-Cube LED Driver (Figure 2K).

## Hardware Architecture

**Figure 3:**
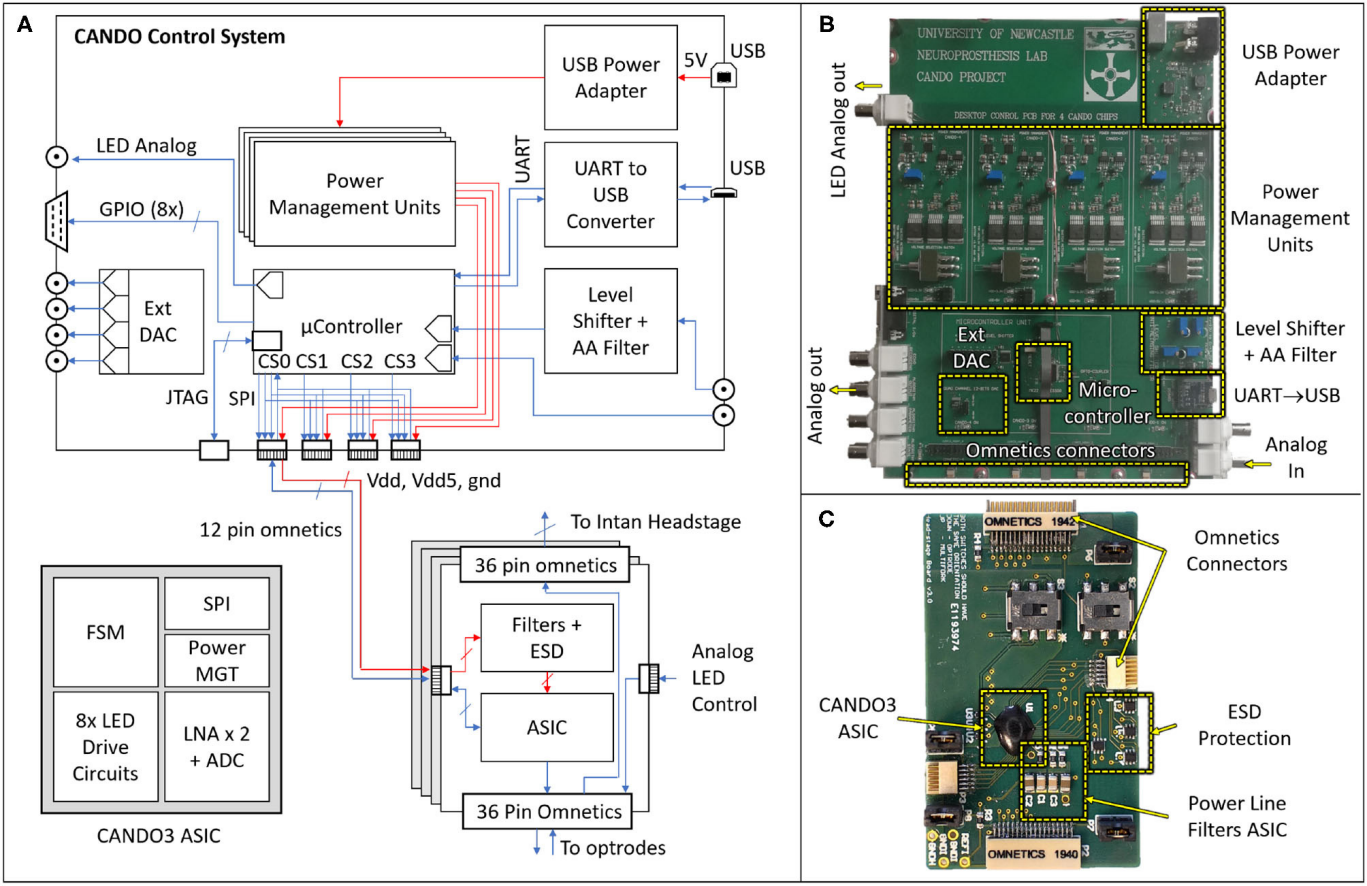
Block diagram **(A)** and physical implementations of the CANDO Control System **(B)** and CANDO Headstage **(C)**. The headstage is based on a CANDO3 Application Specific Integrated Circuit (ASIC) comprising a Finite State Machine (FSM) digital block that controls the LED Drive circuits and Low Noise Amplifiers (LNAs). Communication with the control system is implemented by a Serial Peripheral Interface (SPI). The CANDO-CS is based on an MK22FN512VLH12 Microcontroller Unit (MCU) which contains Analog-to-Digital Converter (ADC), Digital-to-Analog Converter (DAC), SPI, Universal Asynchronous Receiver Transmitter (UART) and General-Purpose Inputs/Outputs (GPIOs) peripherals. There are four power management circuits, a level-shifting circuit for signals being introduced via the Intan Recording Controller, and a second order Anti-Aliasing Filter (AAF) with a cut-off frequency of 250 Hz. There is also a USB-to-UART converter for communication between the MCU and a PC. The Joint Test Action Group (JTAG) Connector allows for programming and debugging the MCU firmware.

The hardware (HW) architecture of the developed system is depicted in Figure 3, showing both the block diagram (left) and physical implementation (right) of the CANDO-CS and CANDO headstage. A detailed description of these components is provided in the following sections.

### CANDO-CS

The control system is based around an ARM Cortex-M4 Microcontroller Unit (MCU). We chose an MK22FN512VLH12 MCU in a 64-way LQFP package, which has a maximum core speed of 120 MHz and 512KB embedded flash and 128KB RAM, due to its low power consumption and DSP processing capabilities. The MCU receives neural data, processes it in real-time through various closed-loop algorithms and sends stimulation commands. Power to the CANDO-CS is provided via a 5 V USB port, which is then regulated by four independent power management circuits, delivering isolated supplies to each of the CANDO headstages. This prevents noise through the power lines from interfering between different ASICs.

The control system has been designed for flexibility with a variety of inputs and outputs to ensure compatibility with existing commercial systems and allow real-time performance evaluation. The input signal (recorded data) can be provided to the MCU as analog input via a BNC connector, or digital input via an Omnetics connector. For the former there is an option to feed the analog input through a level-shifting circuit, which re-scales a ±10 Vpp signal to a signal between 0 and 3.3 Volt. There is also a second order anti-aliasing filter (AAF) at the output of the level-shifting circuit with a cut-off frequency of 250 Hz. For digital input, there are four Omnetics connectors on the board which address power (5 Volt and 3.3 Volt supply lines) and bidirectional communication between the MCU and the CANDO headstages via a Serial Peripheral Interface (SPI). Similarly, the algorithm output signals can be provided in analog via a BNC connector or in digital (SPI) via the omnetics connector. The remaining five BNC and D-type connectors are used for signal monitoring during operation, for example, the recorded input, the algorithm output and analog levels indicating different experimental phases. Due to the MCU being limited to a single DAC peripheral, an LTC2604 external 4-channel DAC was introduced to the system which is also addressed by the MCU via an SPI. Further digital timing signals can be sent via the D-type connector, generated by eight General Purpose Inputs/Outputs (GPIOs) on the MCU. The USB to UART (Universal Asynchronous Receiver Transmitter) circuit allows for bidirectional communication between the MCU and a computer, allowing parameter configuration and data recording via the CANDO GUI.

### CANDO Headstage

The CANDO headstages are based on a custom ASIC (CANDO3), which is an improvement over our previously reported work (Ramezani et al., 2018). This is a System-on-Chip (SoC) consisting of an analogue front-end (AFE) and back-end digital controller. The AFE includes: two (2) differential low noise recording channels each using a cascade of 4 amplification stages (including a 2nd order highpass and 1^*st*^ order lowpass filter) based on Liu et al. (2017); eight (8) stimulation channels each capable of driving a μLED using an H-bridge based drive circuit. The AFE is controlled using a Finite State Machine (FSM) which implements a command interpreter and SPI interface for external communication. The FSM and AFE require a 3.3 V supply, whereas the stimulation circuits require a 5 V supply. All the SPI, power and ground pins are provided via a 12-way Omnetics connector.

There are three more Omnetics connectors on the headstage, two 36-way and one 12-way. One of the 36-way connectors is used to connect to a variety of multielectrode arrays for recording. Two channels from the array are recorded via the CANDO3 ASIC. The remaining electrodes can be recorded simultaneously using an Intan headstage that is connected via the second 36-way connector. Up to six LEDs can be driven via the 12-way connector.

### CANDO Optrode

The CANDO headstage has been designed to drive custom implantable LED optrodes. The example shown in Supplementary Figure 1 comprises of a single penetrating shank 3.0 mm long and 0.3 mm wide, incorporating two gold recording contacts and a micro-LED (TR2227, Cree). The head region has contact pads which connect to an off-the-shelf 6-pin connector with a pitch of 1.27 mm between pins (Supplementary Figure 1C). Other geometries we are developing include forks and multifork arrays (Figure 2D). Supplementary Figure 2 illustrates a fork with four shanks 5.0 mm long and 0.3 mm wide. The recording contacts are 240 μm x 100 μm stadium shapes and exhibit impedances between 8 and 10 k Ω at 1 kHz. The light output of the LED was measured using a polymer integrating sphere (SP50, Artifex) to be between 0.41 and 0.53 mW at a current of 1 mA. Data for recording impedance and light output were initially characterised over at least 10 optrodes, and the characteristics of subsequent devices routinely fall within these ranges.

Optrodes are fabricated from 200 μm silicon wafers. First the wafers undergo a standard solvent clean procedure in n-methyl pyrrolidine (NMP) followed by isopropanol (IPA) and rinsing deionised water. A 1 μm-thick insulation SiO_2_ layer is deposited by chemical vapour deposition on the silicon surface. Ti/Au/Ti metallisation is deposited by evaporation on top of the insulation, and patterned by UV photolithography to outline the metal tracks. Metal patterning is carried out by a selective wet etch based on NH_4_OH:H_2_O_2_ (1:2) for titanium, and K_3_Fe(CN)_6_, Na_2_S_2_O_3_ and CS(NH_2_)_2_ in deionised water mixture for gold. The Ti/Au/Ti patterns are then capped with a second SiO_2_ insulation layer. Contact windows through the top insulation needed to access the bonding pads and LED sites are opened by reactive ion etch (RIE), using a Ti/Ni metal mask which is deposited by e-beam evaporation and patterned by UV photolithography and wet etching. Optrode singulation is achieved by deep reactive ion etching (DRIE). The LED contact pads are prepared by Au electroplating, and micro-LEDs (TR2227, Cree) are placed on gold LED pads on the optrode shaft and bonded thermosonically using a pick-and-place Fineplacer Lambda tool by applying a 1 s energy pulse of 300 mW at 120°C. The optrode surface is then prepared for encapsulation by a solvent clean in acetone and isopropanol in an ultrasonic bath, then rinsed in deionised water. The optrode and LED are encapsulated with a 20 μm-thick parylene-C conformal layer using a PSD2010 Labcoater from Speciality Coating Systems.

## Firmware

### Firmware Overview

**Figure 4:**
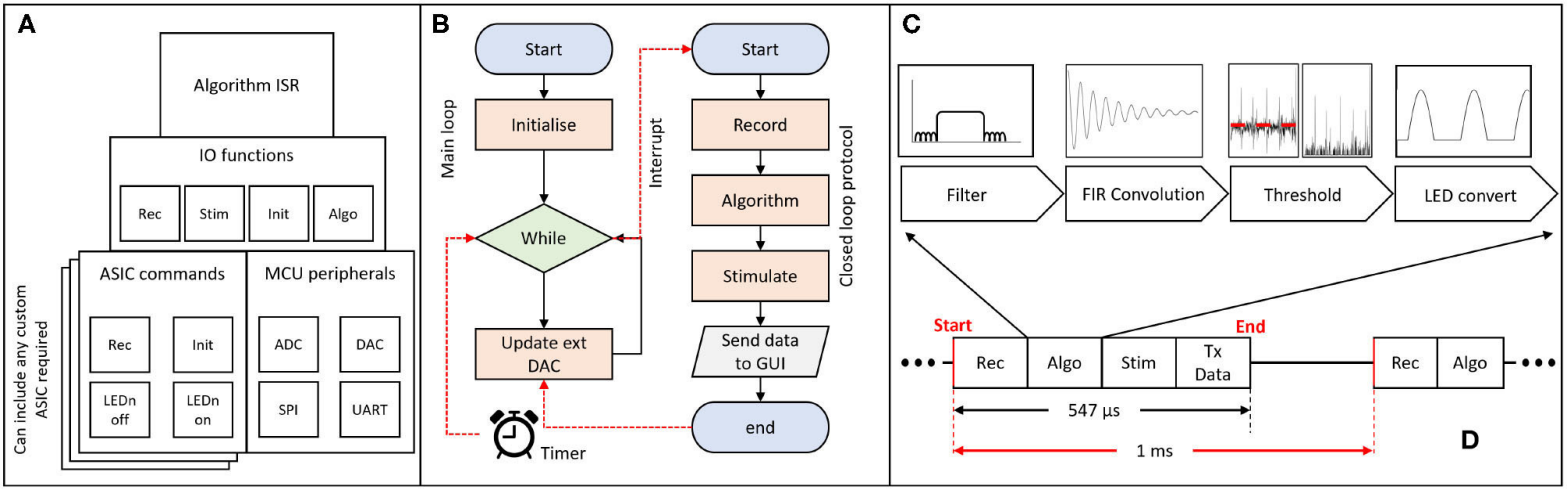
Schematic of firmware implementation of closed-loop algorithms; **(A)** block diagram representation of the firmware architecture; the algorithm Interrupt Service Routine (ISR) determines the program flow and utilises functions from the Input/Output (IO) layer. The ASIC commands block allows for different drivers to be introduced according to the desired ASIC; different layers represent alternative commercial/custom systems. The main external/internal communication protocols are addressed in the MCU peripherals block; **(B)** flowchart of the main method and ISR; the main method is responsible for performing the code initialisation and control of the external DAC. The ISR is triggered every 1 ms and performs the recording, algorithm processing, stimulation and data transmission over UART. The program then returns to the main method. **(C)** block diagram representation of one inbuilt algorithm for phase-shifting neural oscillations via an FIR filter. **(D)** ISR execution time (no more than 547 μs) for a single ASIC being controlled (recording and stimulation) by the MCU and for the data to be processed and transmitted to the PC via UART.

The firmware on the MCU is responsible for controlling the hardware and performing the closed-loop processing. The overall code architecture is summarised in Figure 4A. The top layer contains the code executed in an Interrupt Service Routine (ISR), which provides time accuracy for the algorithm execution. This is turn can utilise function from the IO functions layer. The IO functions act as a wrapper library to provide control over the stimulation and recording capabilities of commercial/custom equipment. The ASIC commands layer contains a custom driver for addressing the CANDO3 ASIC. If an alternative custom ASIC is required then an appropriate driver would need to be included. By including the CANDO3 ASIC driver code we intend to provide a template to enable streamlined development of custom drivers. The MCU peripherals layer provides control over external/ internal communication, and allows for analogue signals to be sent or received by the MCU.

There are two main parts in the firmware: a) the main.c file and b) the algorithm Interrupt Service Routine (ISR). Figure 4B provides a simplified flowchart for both parts. A more detailed flowchart can be found in the Supplementary Figure 3. The main file is where all initialisations take place, including the ISR initialisation. In the main loop, if the start button on the CANDO GUI has been pressed, the on/off state, phase and output of the algorithm values are updated via the external DAC. Otherwise, the external DAC is powered down.

For the algorithm ISR, the period of interrupt has been set to 1 ms to provide timing accuracy while allowing a sufficient sampling frequency for LFP recordings which typically contain brain oscillations at frequencies less than 100 Hz. A 1ms interrupt allows for the effective sampling frequency per recording channel on the ASIC to be 500 Hz (data per channel received in an alternating manner). Once the ISR is entered, the MCU will execute the record function where, based on the input of the user, it receives a value either from a CANDO headstage via SPI communication or from a data acquisition system via one of the ADC channels on the MCU. Once the recorded value has been obtained, the code checks for a series of user defined parameters, such as the start button, on/off cycle duration and type of stimulation, which are originating from the GUI. Depending on what the user has selected the program will execute the algorithm, perform a stimulation command and update algorithm parameters. Finally, at the end of the ISR the recorded signal, algorithm output and parameters are transmitted to the PC via a UART interface for visualisation and storage. The receive UART buffer is also checked in order to decode the data packets received by the GUI and execute the corresponding commands on the MCU side. For each ASIC, the execution time of the MCU to perform the recording, algorithm processing, stimulation and data transmission (PC via UART) was measured to be equal to no more than 547 μs, as shown in Figure 4D.

### Closed-Loop Algorithms

The system we describe in this paper has been designed to test the effect of open- and closed-loop optogenetic stimulation of neural tissue. The software provides parameter selection options to create and modulate stimulation profiles, for example the timing and intensity open-loop stimulus trains, or determining how real-time recordings are converted into closed-loop stimulation. Our closed-loop algorithms are based around a finite impulse response (FIR) convolution which band-pass filters and phase-shifts oscillatory LFP signals. Supplementary Figure 4A provides an example of the kernel of the FIR filter for different phase-shifts (0–315°), to illustrate the effect of the phase-shift on the stimulation profile. The corresponding gain and phase responses of the FIR filter kernels are also illustrated in Supplementary Figures 4B,C, respectively. The interface allows periods of closed-loop stimulation with a variety of parameter settings (e.g., different phase-shifts) to be delivered in pseudorandomised order, interspersed with control periods of no stimulation. We include documentation to aid users to implement further custom algorithms in the user manuals in the Supplementary Material.

## Software

**Figure 5:**
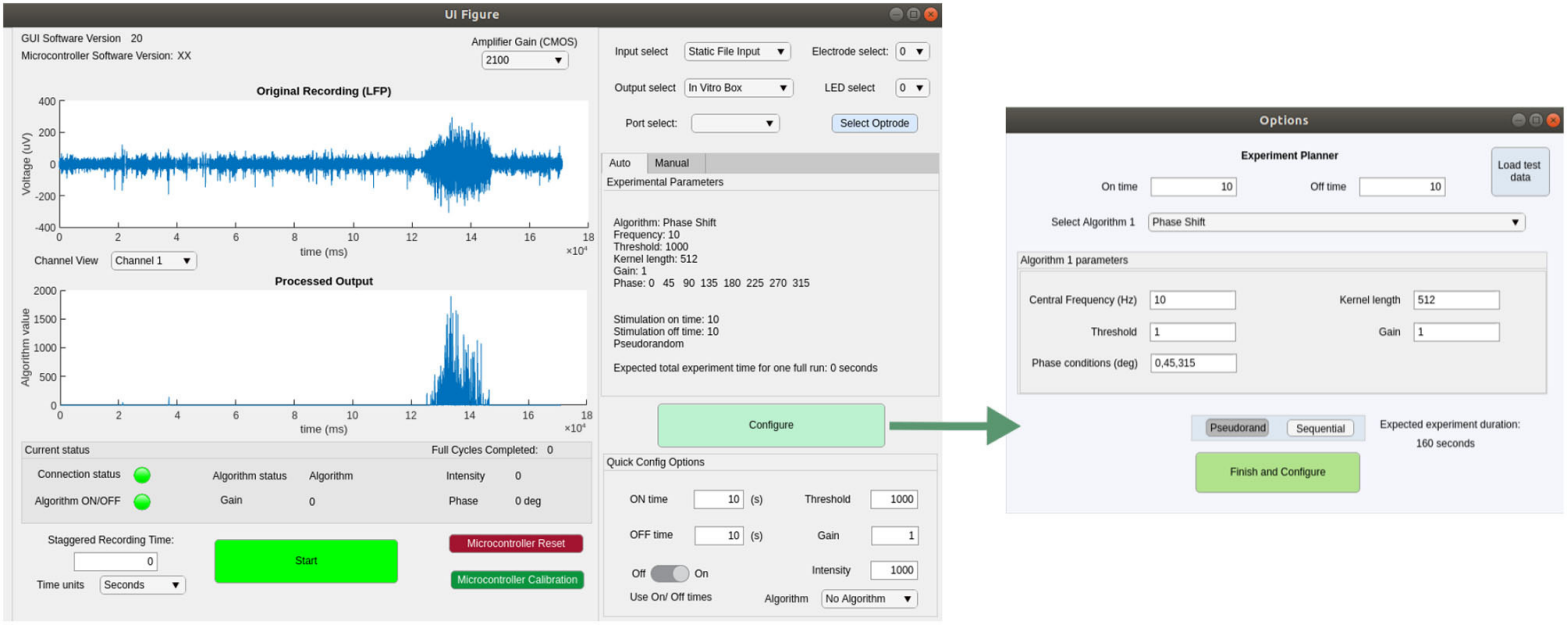
Screenshots showing the main functionality of the GUI for configuring and controlling closed- or open-loop stimulation experiments.

We implemented a GUI in Matlab 2019b, compatible with all major operating systems and tested on Windows 10, OSX and Linux (Ubuntu 18.04.5 LTS). This software is able to detect a connected serial device and send signals to interact with the MCU firmware. Figure 5 shows screenshots of the main GUI windows, which allow the experimenter to control recording, stimulation and closed-loop algorithm parameters. The Experiment Planner window allows the user to select one or more closed-loop algorithms and associated parameters, such as the frequency and phase-shift of the FIR filter and the gain of LFP-to-light conversion. Multiple algorithms and parameter ranges can be selected, to be delivered in sequential or pseudo-randomised order, allowing their effect on the neural tissue to be compared. There are additional “quick config” options to allow a user to choose the duration of each closed-loop stimulation epoch, as well as of interspersed control periods with no stimulation. For testing and configuration purposes, the software is also able to read from pre-recorded data files and calculate and plot expected stimulation output, to allow a user to determine appropriate parameters in advance. All source code and the latest compiled version of this software can be found at our github repository.^3^ For further details please see the user guide in the Supplementary Material.

## Stability Testing

Performance of the hardware, firmware and software operation was validated on the bench prior to use in *in-vitro* and *in-vivo* experiments. In this case the CANDO headstage was not part of the testing. Only the CANDO-CS, with the corresponding firmware version, and the CANDO GUI were used. The system was left to operate for a period of 24 h (test duration) without intervention with a 1 Vpp, 10 Hz sine-wave input provided by a Keithley 33500B Waveform Generator via the ADC. The signal was processed by a closed-loop algorithm providing output that is shown in the Supplementary Figure 5A. Spectral analysis—Supplementary Figure 5B—shows the dominant frequency component of the recorded signal at 10 Hz. The stability of the interrupt period and ISR execution time were also investigated, with the former being 1 ms with a ±13 μs deviation (Supplementary Figure 5C) and the latter occupying approximately 55% of the ISR period (Supplementary Figure 5D).

## Results

### Simultaneous Recording With CANDO and Intan Headstages

**Figure 6:**
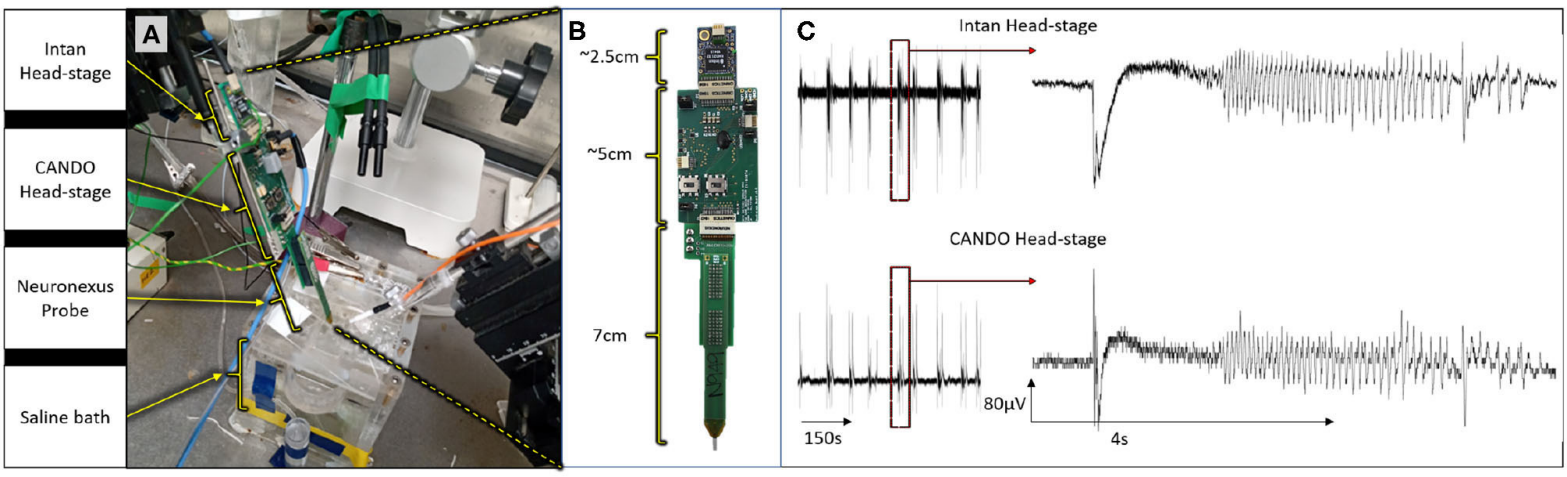
Results from an *in-vitro* experiment on a mouse brain slice; **(A)** experimental set-up, in which both the Intan headstage and CANDO headstage are connected to different recording sites of a single 64-channel NeuroNexus probe; **(B)** dimensions of the Intan headstage, CANDO headstage and NeuroNexus probe; **(C)** experimental results show simultaneous recording from the Intan headstage (top) and CANDO headstage (bottom) of a seizure-like event elicited by 4-AP.

All animal experiments were approved by the local ethics committee at Newcastle University and performed under appropriate UK Home Office licenses in accordance with the Animals (Scientific Procedures) Act 1986. We first validated the recording capabilities of our system by recording simultaneously with both the CANDO headstage and a commercial Intan headstage during an *in-vitro* experiment (C57BL/6J mouse brain slice). Both headstages were connected to different recording sites on a 64-Channel NeuroNexus probe (Figure 6A). The Intan headstage, CANDO headstage and NeuroNexus probe had lengths of 2.5 cm, 5 cm and 7 cm respectively, accumulating to a total of 14.5 cm (Figure 6B). Two recording sites were connected to the CANDO3 ASIC front-end electronics and six more recording sites were connected to the Intan headstage. The slice was perfused with ACSF-containing 4-Aminopyridine (4-AP) (100 μM) to induce seizure-like events. The Intan headstage was connected to an Intan Recording Controller and the a signal channel was also passed to a Micro1401-4 Data Acquisition System along with the recorded signal from the CANDO headstage. Figure 6C illustrates the recorded activity by both the Intan headstage (top) and CANDO headstage (bottom). Examples of seizure-like activity are expanded on the right-hand side of the figure.

### Closed-Loop Optogenetic Stimulation *in-vitro*

**Figure 7:**
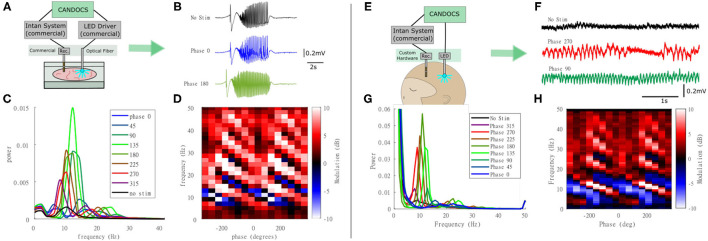
Closed-loop optogenetic stimulation *in-vitro* (mouse brain slice) and *in-vivo* (non-human primate). **(A)** Schematic of the *in-vitro* experimental set-up, in which optogenetic stimulation delivered via an optic fibre is controlled in real-time from LFP recordings; **(B)** LFP traces during no stimulation and stimulation during 0° and 180° phase shift; **(C)** Power spectra for each of the phase conditions; **(D)** Modulation of power relative to no stimulation epochs at each frequency for the different phase conditions revealing a phase-dependent enhancement (red) and suppression (blue) of oscillations around 10 Hz. Note that phase has been unwrapped and plotted over two cycles to reveal the pattern more clearly. **(E)** Schematic of the *in-vivo* experimental set-up in which closed-loop optogenetic stimulation is delivered to the brain of an anesthetised animal via an implanted optrode; **(F)** LFP traces during no stimulation and stimulation during 270° and 90° phase shift; **(G)** Power spectra for each of the phase conditions; **(H)** Power at each frequency for the different phase conditions revealing a phase-dependent enhancement (red) and suppression (blue) of oscillations around 10 Hz.

Next, we used the CANDO-CS for closed-loop control of optogenetic stimulation *in-vitro*. This experiment used a brain slice from a transgenic mouse expressing an excitatory opsin in pyramidal neurons (EMX-ChR2, crossbred from Stock #005628 and Stock #12569, Jackson Laboratory). Seizure-like activity was again elicited with bath-application of 4-AP. A diagram of the experimental set-up is illustrated in Figure 7A. This example demonstrates use of the CANDO-CS in combination with commercial components typically used in an *in vitro* laboratory rig. LFPs and action potentials were acquired using a 32-Channel NeuroNexus probe and 32-channel Intan headstage. One channel of LFP was relayed to the CANDO-CS via the internal ADC of the MCU. Closed-loop stimulation with a 10 Hz FIR filter frequency and different phase-shifts between 0°-315° with a 45° step were delivered in pseudorandomised epochs with an on/off time of 200 s/100 s. The output of the algorithm was sent to a T-Cube LED driver via the internal DAC of the MCU. The LED driver was connected to an optical fibre that deliver the optical stimulation to the slice. Figure 7B shows example LFP recordings with no stimulation and closed-loop stimulation with a phase shift of 0° and 180°. The power spectra during periods of closed-loop optogenetic stimulation (Figure 7C) reveal that some phase-shifts drove oscillations through positive feedback. Figure 7D plots the power at each frequency, relative to no stimulation epochs, for the different phase-shift conditions. A characteristic pattern of phase-dependent enhancement (red) and suppression (blue) of oscillations around 10 Hz is observed.

### Closed-Loop Optogenetic Stimulation *in-vivo*

The CANDO-CS and CANDO-optrodes were also used to deliver closed-loop optogenetic stimulation *in-vivo* in an anethetised non-human primate that had previously been injected with a virus to express an excitatory opsin under a pan-neuronal promoter (AAV8-hSyn-Chronos-GFP, University of North Carolina Vector Core, USA). Figure 7E shows a schematic of the experimental set-up. An Intan headstage recorded brain signals from a linear multielectrode array (260 μm shaft diameter, 32-channels, 100 mm spacing, U-probe, Plexon Inc). The recorded signals were acquired by an Intan Recording controller. The CANDO-CS received one channel of LFP from the Intan Recording controller and performed the closed-loop algorithm (filtering at 10 Hz, followed by a phase-shift) with an on/off time and phase conditions set to 10 s/10 s and 0°-315° with a 45° step, respectively. The output of the algorithm was sent to the CANDO headstage, which in turn drove 5 LEDs on a CANDO fork optrode. Figure 7F shows LFP traces for the case of no stimulation and stimulation with a phase of 270° and a phase of 90°. As in the *in-vitro* experiments, the power spectra during periods of closed-loop optogenetic stimulation (Figure 7G) reveal that some phase-shifts drove oscillations through positive feedback. Figure 7H again plots the power at each frequency, relative to no stimulation epochs, for the different phase-shift conditions, revealing a phase-dependent enhancement (red) and suppression (blue) of oscillations around 10 Hz. For more details on the neurophysiological effects and applications of closed-loop optogenetic stimulation, see (Zaaimi et al., 2020).

## Discussion and Future Directions

In this paper we have described the design and implementation of a system for closed-loop optogenetic stimulation, and provided example uses in ongoing *in-vitro* and *in-vivo* experiments. The system incorporates custom hardware, firmware and software and is also compatible with a variety of commercial components. The system architecture distributes functionality across multiple technologies so as to optimise performance. For example, the front-end recording/stimulation headstage is implemented on a custom ASIC to allow low-power amplifiers to be placed on/near the optrodes to minimise noise, while the closed-loop algorithm is implemented on a microcontroller to ensure precise real-time control, and a desktop computer GUI provides user-friendly configuration and visualisation of experiments. In future, we envisage the system being translated toward a fully implantable device suitable for human use, with these components providing the basis for a brain implant, subcutaneous control unit and external configuration. Realising such a device would require further work to implement wireless power and communication, as well as establishing the long-term encapsulation of active electronics within the nervous system (see Lamont et al., 2021). In the meantime, our system provides a platform for exploring the effects of closed-loop optogenetic stimulation on neural dynamics and behaviour in a variety of preclinical models.

At present we have implemented relatively simple closed-loop algorithms involving an FIR convolution to filter and phase-shift the neural signal. We are currently exploring how these simple algorithms can be used to impose positive or negative feedback loops to enhance or suppress oscillations at specific frequencies. However, the system has the capability to implement more sophisticated algorithms, for example using a phase-locked loop to track oscillations that vary in frequency (Lo and Lee, 2000). We have provided source code and user guide to assist with implementation of new algorithms, although this requires familiarity with Embedded C for updating the firmware on the MCU. In addition, our current algorithms are single-input single-output, but the architecture has been designed in future to allow multiple-input multiple-output (MIMO) transformations such that asynchronous stimulation through multiple LEDs could be controlled in real-time by spatio-temporal activity patterns in neural tissue. For each ASIC a total time of no more than 547 μs is required by the MCU to record (33 μs), process (428 μs), stimulate (55 μs) and transmit the data (27 μs); the remaining 3 μs originate from branch statements in the code. We are currently working on optimising the processing part in order to significantly reduce the computational time required per sample. Some initial efforts have allowed for the algorithm processing time to be reduced to approximately 385 μs. Further optimisation, in order to minimise processing time, will be necessary for translation to a MIMO architecture. In future we would also like to incorporate control signals with higher bandwidth, in particular, the spiking activity of individual neurons. Detecting, discriminating and sorting spike waveforms is a computationally-expensive and power-intensive process, but in parallel to the current work we are developing low-power front-end hardware to simplify this problem by detecting and sorting spikes at source using template-matching (Luan et al., 2018). Taken together, the co-design of custom integrated circuits, embedded systems and hardware/software architectures for closed-loop control, combined with the power of optogenetics to deliver targeted stimulation without interfering with concurrent electrophysiological recording, will enable a new generation of spatio-temporal and cell-type specific bidirectional interfaces with the nervous system.

## Data Availability Statement

The original contributions presented in the study are included in the article/Supplementary Material, further inquiries can be directed to the corresponding author/s.

## Author Contributions

DF: conceptualisation, hardware design, firmware development, algorithm implementation, system integration, testing, writing—original draft, writing—review and editing, and visualisation. FH: conceptualisation, software development, algorithm implementation, testing, writing—original draft, writing—review and editing, and visualisation. RT: hardware design. DW: *in-vitro* and *in-vivo* testing. MT: *in-vitro* and *in-vivo* testing. EE-C: optrode fabrication, system integration, writing—original draft, and writing—review and editing. RB and JG: optrode fabrication, writing—original draft, writing—review, and editing. AP: optrode fabrication. DH and YL: ASIC design. TC: ASIC design supervision, writing—review and editing. AO'N: optrode fabrication supervision. MK: algorithm implementation supervision and software supervision. PD: conceptualisation, hardware design supervision, firmware development supervision, algorithm implementation supervision, system integration supervision, ASIC design supervision, writing—review and editing, and visualisation. AJ: conceptualisation, algorithm implementation supervision, *in-vitro* and *in-vivo* testing supervision, writing—review and editing, and visualisation. All authors contributed to the article and approved the submitted version.

## Funding

This work was supported by the Welcome Trust foundation, 102037/Z/13/Z, and the EPSRC, NS/A000026/1.

## Conflict of Interest

The authors declare that the research was conducted in the absence of any commercial or financial relationships that could be construed as a potential conflict of interest.

## Publisher's Note

All claims expressed in this article are solely those of the authors and do not necessarily represent those of their affiliated organizations, or those of the publisher, the editors and the reviewers. Any product that may be evaluated in this article, or claim that may be made by its manufacturer, is not guaranteed or endorsed by the publisher.

## Footnotes

^1^ https://github.com/NewcastleRSE/CANDO-CS

^2^ http://www.cando.ac.uk/aboutcando/

^3^ https://github.com/NewcastleRSE/CANDO-CS

## References

[B1] Bourget D. Bink H. Stanslaski S. Linde D. Arnett C. Adamski T.. (2015). An implantable, rechargeable neuromodulation research tool using a distributed interface and algorithm architecture, in 7th International IEEE/EMBS Conference on Neural Engineering (NER) (Montpellier), 61–65. 10.1109/NER.2015.7146560

[B2] Bouthour W. Megevand P. Donoghue J. Luscher C. Birbaumer N. Krack P. (2019). Author correction: biomarkers for closed-loop deep brain stimulation in Parkinson disease and beyond. Nat. Rev. Neurol. 15:363. 10.1038/s41582-019-0189-x 30992557

[B3] Buzsaki G. Anastassiou C. Koch C. (2012). The origin of extracellular fields and currents—EEG, ECOG, LFP and spikes. Nat. Rev. Neurosci. 13, 407–420. 10.1038/nrn3241 22595786 PMC4907333

[B4] Cagnan H. Denison T. McIntyre C. Brown P. (2019). Emerging technologies for improved deep brain stimulation. Nat. Biotechnol. 37, 1024–1033. 10.1038/s41587-019-0244-6 31477926 PMC6877347

[B5] Deisseroth K. (2011). Optogenetics. Nat. Methods 8, 26–29. 10.1038/nmeth.f.324 21191368 PMC6814250

[B6] Fisher R. Boas W. Blume W. Elger C. Genton P. Lee P.. (2005). Epileptic seizures and epilepsy: definitions proposed by the International League Against Epilepsy (ILAE) and the International Bureau for Epilepsy (IBE). Epilepsia 46, 470–472. 10.1111/j.0013-9580.2005.66104.x 15816939

[B7] Frank J. A. Antonini M.-J. Anikeeva P. (2019). Next-generation interfaces for studying neural function. Nat. Biotechnol. 37, 1013–1023. 10.1038/s41587-019-0198-8 31406326 PMC7243676

[B8] Gagnon-Turcotte G. Bilodeau G. Tsiakaka O. Gosselin B. (2020). Smart autonomous electro-optic platforms enabling innovative brain therapies. IEEE Circ. Syst. Mag. 20, 28–46. 10.1109/MCAS.2020.3027220

[B9] Jackson A. Moritz C. Mavoori J. Lucas T. Fetz E. (2006). The neurochip BCI: towards a neural prosthesis for upper limb function. IEEE Trans. Neural Syst. Rehabil. Eng. 14, 187–190. 10.1109/TNSRE.2006.875547 16792290

[B10] Kinney J. P. Bernstein J. G. Meyer A. J. Barber J. B. Bolivar M. Newbold B.. (2015). A direct-to-drive neural data acquisition system. Front. Neural Circ. 9:46. 10.3389/fncir.2015.00046 26388740 PMC4555017

[B11] Kokkinos V. Sisterson N. Wozny T. Richardson R. (2019). Association of closed-loop brain stimulation neurophysiological features with seizure control among patients with focal epilepsy. JAMA Neurol. 76, 800–808. 10.1001/jamaneurol.2019.0658 30985902 PMC6583077

[B12] Lamont C. Grego T. Nanbakhsh K. Idil A. S. Giagka V. Vanhoestenberghe A.. (2021). Silicone encapsulation of thin-film SiOx, SiOxNy and SiC for modern electronic medical implants: a comparative long-term ageing study. J. Neural Eng. 18:055003. 10.1088/1741-2552/abf0d6 33752188 PMC8208634

[B13] Liu Y. Luan S. Williams I. Rapeaux A. Constandinou T. G. (2017). A 64-channel versatile neural recording SOC with activity-dependent data throughput. IEEE Trans. Biomed. Circ. Syst. 11, 1344–1355. 10.1109/TBCAS.2017.2759339 29293425

[B14] Liu Y. Urso A. Martins da Ponte R. Costa T. Valente V. Giagka V.. (2020). Bidirectional bioelectronic interfaces: system design and circuit implications. IEEE Solid State Circ. Mag. 12, 30–46. 10.1109/MSSC.2020.2987506

[B15] Lo P.-C. Lee Y.-Y. (2000). Applicability of phase-locked loop to tracking the rhythmic activity in EEGs. Circ. Syst. Signal Process. 19, 171–186. 10.1007/BF01204572

[B16] Lozano A. Lipsman N. Bergman H. Brown P. Chabardes S. Chang J.. (2019). Deep brain stimulation: current challenges and future directions. Nat. Rev. Neurol. 15, 148–160. 10.1038/s41582-018-0128-2 30683913 PMC6397644

[B17] Luan S. Williams I. Maslik M. Liu Y. De Carvalho F. Jackson A.. (2018). Compact standalone platform for neural recording with real-time spike sorting and data logging. J. Neural Eng. 15:046014. 10.1088/1741-2552/aabc23 29623905

[B18] Mickle A. D. Won S. M. Noh K. N. Yoon J. Meacham K. W. Xue Y.. (2019). A wireless closed-loop system for optogenetic peripheral neuromodulation. Nature 565, 361–365. 10.1038/s41586-018-0823-6 30602791 PMC6336505

[B19] Newman J. Zeller-Townson R. Fong M.-F. Arcot Desai S. Gross R. Potter S. (2013). Closed-loop, multichannel experimentation using the open-source neurorighter electrophysiology platform. Front. Neural Circ. 6:98. 10.3389/fncir.2012.00098 23346047 PMC3548271

[B20] Pashaie R. Baumgartner R. Richner T. J. Brodnick S. K. Azimipour M. Eliceiri K. W.. (2015). Closed-loop optogenetic brain interface. IEEE Trans. Biomed. Eng. 62, 2327–2337. 10.1109/TBME.2015.2436817 26011877

[B21] Putzeys J. Raducanu B. C. Carton A. De Ceulaer J. Karsh B. Siegle J. H.. (2019). Neuropixels data-acquisition system: a scalable platform for parallel recording of 10 000+ electrophysiological signals. IEEE Trans. Biomed. Circ. Syst. 13, 1635–1644. 10.1109/TBCAS.2019.2943077 31545742

[B22] Ramezani R. Liu Y. Dehkhoda F. Soltan A. Haci D. Zhao H.. (2018). On-probe neural interface asic for combined electrical recording and optogenetic stimulation. IEEE Trans. Biomed. Circ. Syst. 12, 576–588. 10.1109/TBCAS.2018.2818818 29877821

[B23] Raza C. Anjum R. ul Ain Shakeel N. (2019). Parkinson's disease: mechanisms, translational models and management strategies. Life Sci. 226, 77–90. 10.1016/j.lfs.2019.03.057 30980848

[B24] Sahel J.-A. Boulanger-Scemama E. Pagot C. Arleo A. Galluppi F. Martel J. N.. (2021). Partial recovery of visual function in a blind patient after optogenetic therapy. Nat. Med. 27, 1223–1229. 10.1038/s41591-021-01351-4 34031601

[B25] Schalk G. McFarland D. Hinterberger T. Birbaumer N. Wolpaw J. (2004). BCI2000: a general-purpose brain-computer interface (BCI) system. IEEE Trans. Biomed. Eng. 51, 1034–1043. 10.1109/TBME.2004.827072 15188875

[B26] Shen Y. Campbell R. E. Côté D. C. Paquet M.-E. (2020). Challenges for therapeutic applications of opsin-based optogenetic tools in humans. Front. Neural Circ. 14:41. 10.3389/fncir.2020.00041 32760252 PMC7373823

[B27] Siegle J. Lopez A. Patel Y. Abramov K. Ohayon S. Voigts J. (2017). Open Ephys: an open-source, plugin-based platform for multichannel electrophysiology. J. Neural Eng. 14:045003. 10.1088/1741-2552/aa5eea 28169219

[B28] Steinmetz N. A. Aydin C. Lebedeva A. Okun M. Pachitariu M. Bauza M.. (2021). Neuropixels 2.0: a miniaturized high-density probe for stable, long-term brain recordings. Science 372:6539. 10.1101/2020.10.27.358291 33859006 PMC8244810

[B29] Vazquez-Guardado A. Yang Y. Bandodkar A. J. Rogers J. A. (2020). Recent advances in neurotechnologies with broad potential for neuroscience research. Nat. Neurosci. 23, 1522–1536. 10.1038/s41593-020-00739-8 33199897

[B30] Wang Y. Hutchings F. Kaiser M. (2015). Computational modeling of neurostimulation in brain diseases. Prog. Brain Res. 222, 191–228. 10.1016/bs.pbr.2015.06.012 26541382

[B31] Yang Y. Wu M. Vazquez-Guardado A. Wegener A. J. Grajales-Reyes J. G. Deng Y.. (2021). Wireless multilateral devices for optogenetic studies of individual and social behaviors. Nat. Neurosci. 24, 1035–1045. 10.1038/s41593-021-00849-x 33972800 PMC8694284

[B32] Zaaimi B. Turnbull M. Hazra A. Wang Y. de Souza C. G. Escobedo-Cousin E.. (2020). Closed-loop optogenetic control of normal and pathological network dynamics. Research Square [Preprint]. 10.21203/rs.3.rs-78230/v1

[B33] Zelmann R. Paulk A. C. Basu I. Sarma A. Yousefi A. Crocker B.. (2020). Closes: a platform for closed-loop intracranial stimulation in humans. Neuroimage 223:117314. 10.1016/j.neuroimage.2020.117314 32882382 PMC7805582

[B34] Zhou A. Santacruz S. R. Johnson B. C Alexandrov G. Moin A.. (2019). A wireless and artefact-free 128-channel neuromodulation device for closed-loop stimulation and recording in non-human primates. Nat. Biomed. Eng. 3, 15–26. 10.1038/s41551-018-0323-x 30932068

