# Supplementary material for: A Closed-Loop Optogenetic Platform
Source: Front Neurosci. 2021 Sep 10;15:718311. doi: 10.3389/fnins.2021.718311 (PMC8462298; doi:10.3389/fnins.2021.718311)
Supplement: Supplementary file 1 [file Data_Sheet_1.PDF]

CANDO Control System

Frances Hutchings, Dimitris Firfilionis

May 31, 2021

The CANDO Project
Newcastle University

This document contains information about the CANDO (Controlling Abnormal Network Dynamics using Optogenetics) project control system: CANDO-CS. The system consists of a graphical user interface (GUI) installed on a computer and a microcontroller with custom firmware for interacting with recording and stimulating devices. The graphical user interface (GUI) for the CANDO-CS has been developed in MATLAB and connects via a serial port connection to the microcontroller in the *in vitro* control box. The microcontroller firmware has been developed in embedded C and can be compiled and flashed from ahost computer. The GUI allows users to set up experiments and pass a range of parameters to the microcontroller, alongside saving parameter metadata and input from the microcontroller for streamlining analysis.

The following pages include a visual summary of the GUI functionality and a list of parameters and guides for using and further extending the CANDO-CS. The code for the GUI and firmware can be found in our github repository hosted by the Newcastle University Research Software Engineer team.

Contents

1 Parameter list **5**
1.1 Main Interface 5
1.2 Experiment Planner 8

2 Setting up and using the Stimulation Box **11**
2.1 Initial Setup 11
2.2 Example experimental setup 11
2.3 Loading pre-recorded data to test the algorithm output 12
2.4 Setting up a custom algorithm through the UI 13
2.5 Using a test signal from a local machine 13

3 Extending the CANDO-CS **15**
3.1 Extending the user interface 15
3.2 Adding new algorithms to firmware 16

4 Additional Features **16**

5 Analysing results **17**

List of Figures

1 Overview schematic of the GUI functionality and underlying processes . . 4
2 Screen capture of the main GUI window with numbers corresponding the main text information about different key aspects of the user interface. . 5
3 Screen capture of the pop-up Experiment Planner window, with number markers corresponding to the main text information about different parameters and key aspects of this interface. 8
4 Screen Capture of the main GUI showing the manual stimulation options for setting up custom stimulation approaches, with numeric labels corresponding to information in the main text. 14
5 Screen Capture of the Optrode Selector GUI showing the interactable LED and electrode selector for an example optrode device. 18

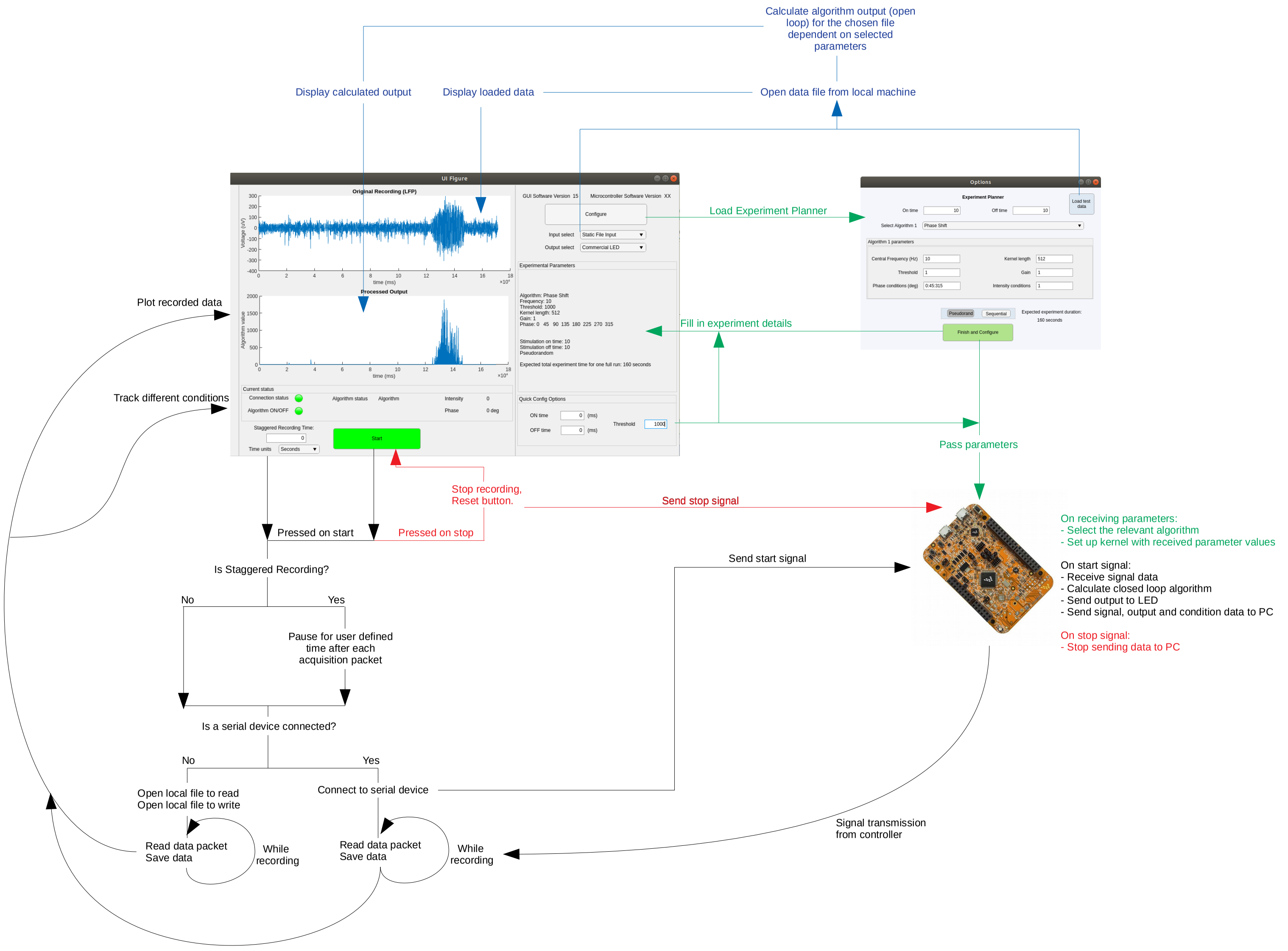

Figure 1: Overview schematic of the GUI functionality and underlying processes

1 Parameter list

1.1 Main Interface

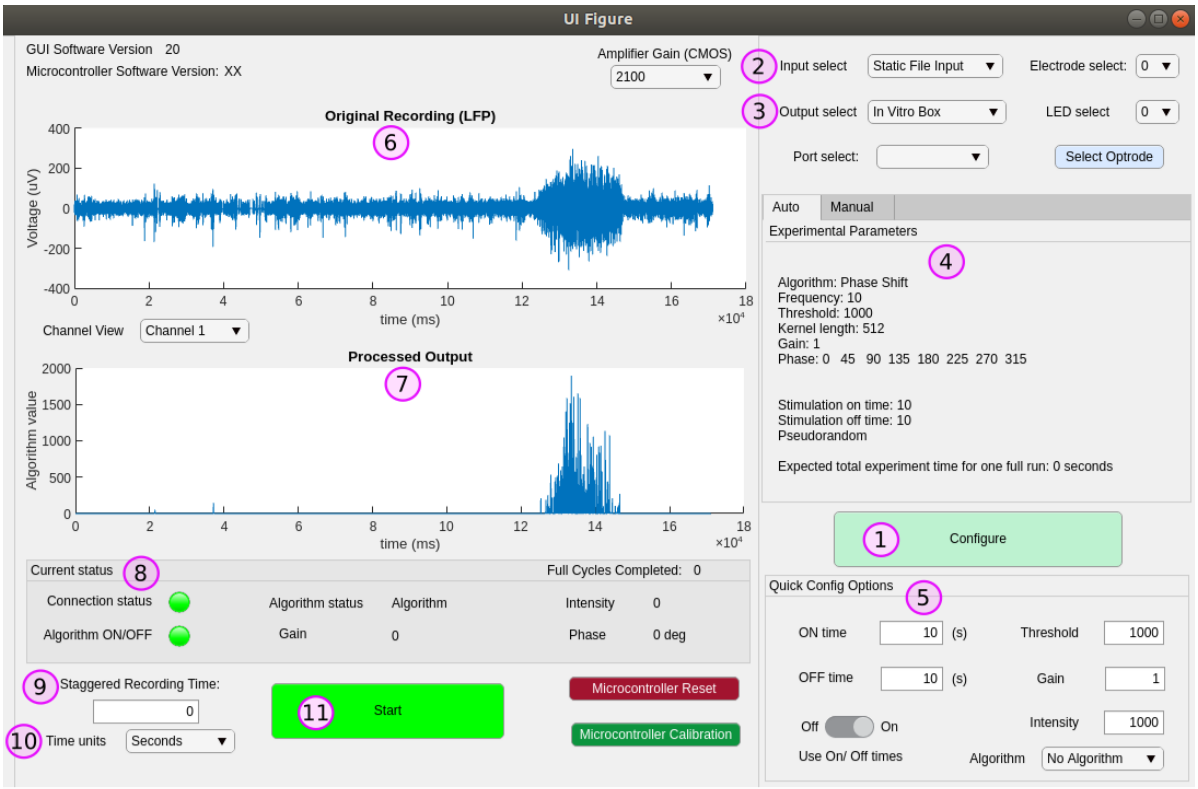

Figure 2: Screen capture of the main GUI window with numbers corresponding the main text information about different key aspects of the user interface.

1. **Configure Button**
   When pressed, this button triggers the appearance of the Experiment Planner window, which is used to input the relevant algorithm parameters. If the start button is pushed without configuring the experimental parameters then no algorithm will be applied, and the expected inputs should be the raw recorded signal from the connected input.

2. **Input select**
   The Input select drop-down box allows a user to choose from preset options for alternative inputs. By default the GUI will attempt to find a serial port connection.

This includes notably the static file input, which will prompt a user to select a file from the host computer. It expects a matlab readable format containing time series data. This data will then be plotted in the Original Recording (LFP) window, and if an algorithm configuration has been chosen then the GUI will call an internal function to calculate an expected Processed Output given the current algorithm parameters. This is intended as a means to determine suitable algorithm parameters before conducting long experiments.

3. **Output select**
   The Output select drop down is intended to pass commands to a connected microcontroller to inform the device what the intended output is to be.

4. **Experimental Parameters window**
   This window is not interactive, and is intended to provide an at-a-glance summary of chosen experimental parameters for users to check and compare. This is initially blank and is populated by the Experimental Planner triggered from the Configure button.

5. **Quick Config Options**
   The Quick Config Options panel contains parameters which are often changed during an experiment, to prevent a user from having to redo the whole experiment setup in the Experiment Planner window. The options are detailed below.

   a) **ON time**
      The On time input box expects a value in ms which translates to the amount of time that the algorithm is on for. When this value is changed, the expected experiment run time is recalculated and a signal is sent to the microcontroller with the updated parameter.

   b) **OFF time**
      The Off time input box expects a value in ms which translates to the amount of time that the algorithm is off for. When this value is changed, the expected experiment run time is recalculated and a signal is sent to the microcontroller with the updated parameter.

   c) **Threshold**
      The threshold value determines the cut off amplitude for the stimulation, where stimulation is only applied if the amplitude is higher than the threshold level. This is best adjusted to ensure that stimulation is only applied when the signal that is intended to be modulated is clearly present, avoiding applying stimulation during background activity. When this value is changed a signal is sent to the microcontroller with the updated parameter.

6. **Original Recording (LFP) graph**
   This set of axis are intended to display the incoming signal from an electrode recording. Plotting the local field potential from the channel that is being used to generate the closed loop stimulation response. This should initialise as blank, and

will update continuously when the start button has been pushed and the GUI is receiving time series data.

7. **Processed Output graph**
   This set of axis are intended to display the algorithm output which will also be directed to the stimulation device. This should correspond to the local field potential signal plotted on the above Original Recording (LFP) graph.

8. **Current Status**
   The current status panel gives live updates as to the current parameter conditions during an experimental run. The different values that are non-interactive are listed below, and are updated according to the signal package from the connected device.

   a) **Connection status**
      This lamp turns green when a connection is made to a serial port device or to an incoming data stream on the local machine.

   b) **Algorithm ON/OFF**
      The Algorithm ON/OFF lamp is intended to indicate when the algorithm is being applied, and when it is not. Most experimental set ups will involve applying a closed loop algorithm for period, then having an off-period for the tissue being stimulated to recover. The lamp will show red when the algorithm is not being applied, and green when it is.

   c) **Algorithm status**
      The Algorithm status is a text label which indicates which algorithm is being used at the current time. This is to allow for experiments to be set up with multiple algorithms being tested in the same experiment. The text label will change as the algorithm changes during the experimental run.

   d) **Intensity**
      The Intensity value is set to zero by default, and this indicator will only change if the Set Intensity algorithm is being used. The intensity parameter is able to vary throughout the experiment to test the impact of different intensity conditions, in this case the intensity value will update as the parameter value changes throughout the experiment.

   e) **Phase**
      The Phase value changes to indicate the current phase condition in the experiment, in degrees.

9. **Staggered Recording Time**
   This option is to facilitate long term recordings, if the box is left blank then when the start button is pressed the GUI is constantly listening for incoming data. When a staggered recording time is inputted the GUI will pause for the designated time period between receiving, plotting and saving 1 second packets of data.

10. **Time units**
    The time units drop down allows a user to indicate the units intended for the staggered recording time.

11. **Start button**
    The Start button triggers recording from the connected device. It initially prompts a a user to select a save file, and then sends a 'start recording' signal to the connected device to trigger the device to send data to the GUI. The incoming data will be plotted on the graph axis and automatically saved along with metadata.

1.2 Experiment Planner

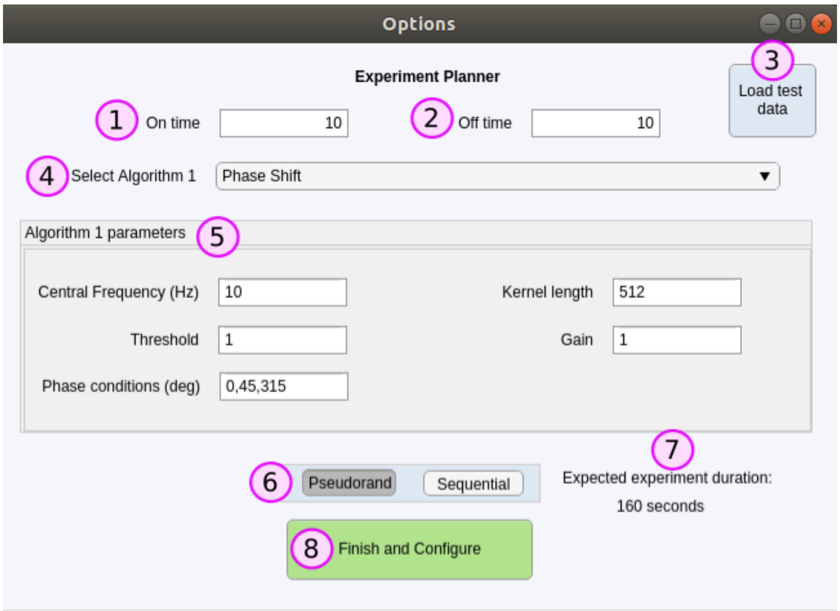

Figure 3: Screen capture of the pop-up Experiment Planner window, with number markers corresponding to the main text information about different parameters and key aspects of this interface.

1. **On time**
   The On time input box expects a value in ms which translates to the amount of time that the algorithm is on for. When this value is changed, the expected experiment run time is recalculated and a signal is sent to the microcontroller with the updated parameter.

2. **Off time**
The Off time input box expects a value in ms which translates to the amount of time that the algorithm is off for. When this value is changed, the expected experiment run time is recalculated and a signal is sent to the microcontroller with the updated parameter.

3. **Load Test Data button**
The Load Test Data button will prompt a user to select a file from the host computer. It expects a matlab readable format containing time series data. This data will then be plotted in the Original Recording (LFP) window, and if an algorithm configuration has been chosen then the GUI will call an internal function to calculate an expected Processed Output given the current algorithm parameters. This is intended as a means to determine suitable algorithm parameters before conducting long experiments.

4. **Select Algorithm 1**
The algorithm select drop-down box allows a user to select between pre-coded algorithms available in the control box. The details of the current algorithms implemented in the control box can be found in the *in vitro* box documentation, and a brief summary of the available options are below.

   a) Phase Shift
   The Phase shift algorithm applies a finite impulse response filter to the incoming signal.

   b) Set Intensity
   The Set Intensity algorithm applies a version of the phase shift algorithm with a preset stimulation level, that is determined by the intensity condition.

5. **Algorithm 1 Parameters**
The algorithm parameters available can change according to the algorithm selected. The Set Intensity and Phase Shift parameters are very similar, the only difference being the Intensity condition that is important for the Set Intensity. This parameter does not impact the Phase Shift algorithm. The parameters are detailed below.

   a) **Central Frequency**
   The central frequency parameter needs to be set to a value that matches the seizure frequency (or the frequency of whatever activity is to be modulated) as closely as possible. The kernel convolution will give the largest values when the frequency of the incoming signal matches the designated central frequency.

   b) **Threshold**
   The threshold value determines the cut off amplitude for the stimulation, where stimulation is only applied if the amplitude is higher than the threshold level. This is best adjusted to ensure that stimulation is only applied when the

signal that is intended to be modulated is clearly present, avoiding applying stimulation during background activity.

c) **Phase conditions**
The Phase condition is the phase in degrees for the algorithm kernel. Experiments have shown that the impact of the algorithm is heavily phase dependent, with some phases reducing seizure duration. Different phases also change the ongoing frequency of the recorded activity. This field is set up so that either a single value can be passed for the phase, or multiple values as a vector in the form of: $lowestvalue : stepsize : uppervalue$.

d) **Kernel length**
The kernel length determines the number of time steps long that the algorithm kernel will be. The default value is 512.

e) **Gain**
The gain value is a multiplier that is applied to the algorithm output to modulate the amplitude of the algorithm produced stimulation signal.

f) **Intensity conditions**
The Intensity parameter sets the amplitude level for the stimulation if the Set Intensity algorithm is used. As with the Phase Shift algorithm the intensity values can be either a single scalar value or a set of values input as a vector in the form $lowestvalue : stepsize : uppervalue$.

6. **Pseudorand / Sequential button selection**
The Pseudorand / Sequential buttons are set to only allow one of the buttons to be pushed at any one time. If the Sequential button is selected then any parameters that vary during the experiment will be stepped through sequentially. If the Pseudorand button is selected then the parameters will be stepped through in a pseudo-random order.

7. **Expected experiment duration**
The Expected experiment duration is calculated based on the number of parameters that need to be stepped through, and the on - off times for the algorithm, to give an estimate of the expected time a single complete run through will take.

8. **Finish and Configure**
The Finish and Configure button closes the experimental planner window and sends the parameter information to the main GUI window, where the details are displayed in the Experimental Parameters window. Pressing this button will also trigger a signal to the microcontroller to indicate the algorithm and number of parameters to expect, followed by sending a data packet with the selected parameter options to the connected device.

2 Setting up and using the Stimulation Box

2.1 Initial Setup

Hardware:

1. Connect a laptop with the user interface installed to the CANDOCS box using a USB cable.
2. Connect the DC shift box to the mains and to the intan output (into input one of the DC shift box)
3. Output 1 of the DC shift box then connects to the ADC
4. DAC port (to the left of the ADC) goes to the LED

Software:

1. Double click on the CANDO user interface (UI) icon if you are using a compiled version
2. If using the mlapp code, open a Matlab instance and then navigate to the folder containing the mlapp file. Double click on the file in the file explorer tab, or alternatively type 'CANDO_control_GUI' in the command window and press enter.
3. Once the UI has loaded it should look like the image in figure 1.

2.2 Example experimental setup

1. Connect the CANDOCS box to a computer with the GUI installed using a USB cable.
2. Open the GUI on the host computer, and select the port which your serial device is connected to in the Port Select dropdown menu. This menu will auto-populate with viable ports.
3. Test the connection: Press the Start Button on the GUI to initialise the connection. The button will change colour to red and change name to 'stop' when the connection is active. A pop-up window will appear asking the user to select a save directory.
4. If the system is properly connected, then the user interface will start recording at this point without applying any stimulation. You should see a trace in the LFP graph. Pressing the start/stop button again will terminate the recording.
5. Press the configure button to trigger the experimental planner window. This will allow you to select an algorithm to apply, and set the parameters for it. The central frequency should match the frequency of ongoing activity that you are interested in

modulating. Default parameters are already entered for each algorithm, and if the defaults are unchanged the selected algorithm will loop through different phases from 0:315 degrees in steps of 45 degrees. This can be useful for ascertaining which phase will suppress or enhance the oscillation of interest - in the case of *in vitro* experiments the distance of the electrode and stimulation source from the slice can impact the phase response.

6. Make sure to enter an 'on time' as the default is zero, so the algorithm will not start until a greater than zero value is entered. This can be changed in the 'Quick Config Options' on the main GUI window if needed.

7. Once algorithm options have been selected, press the 'Finish and Configure' button to pass these parameters to the serial port device and return to the main GUI window. From here, once the 'Start' button is pressed the stimulation should start to be applied and the current values will be plotted on the lower graph. If the Start button is already active then the stimulation should begin as soon as the parameter update is processed by the microcontroller.

8. If you wish to change parameters, the 'Quick Config Options' will allow modifications. Once the recording is in progress the signal and stimulation values will be saved on the host machine in the user chosen save directory. When the stimulation run is completed the data will be saved as matlab readable files. See the section on Analysis for further information about how to process these results.

2.3 Loading pre-recorded data to test the algorithm output

If you have some pre-recorded data then the GUI allows a user to load in and plot the data, and to then visualise an application of an algorithm. Algorithm parameters will be applied over the pre-loaded data.

There are two ways to load in the data:

1. Select the static file input' from the the Input Select drop-down menu (number 2 in figure 2).

2. Press the Configure button (number 1 in figure 2) and then in the Experimental Planner window select 'Load test data' (number 3 in figure 3).

In either case, a file selection window will open up and allow you to select the data to load. Once the data has successfully loaded then it will be plotted in the 'Original Recording (LFP)' graph (number 6 in figure 2).

NB: If you have loaded the data through the Experimental Planner, and there is no serial port connected, then when you press the 'Finish and Configure' button you will be prompted to select a file to write to, read from, and a save file as the UI will expect a test signal from the local machine. Blank files can be used at this point if you do not wish to load a test signal.

Once a file has been loaded, the algorithm parameters can be tested either through the Experimental Planner (launched by the 'Configure' button from the main UI) or using the 'Quick Config Options'. Often the aim of the algorithm parameter tuning is to choose parameters such that the stimulation will be only on / at the maximum when the recorded signal is exhibiting the behaviour that the user wishes to modulate. E.g., in the example plot in figure 2 a time series including a seizure is shown, and parameters have been chosen so that the stimulation is only on during the seizure period.

2.4 Setting up a custom algorithm through the UI

The inbuilt algorithms accessed via the experimental planner are designed to provide a stimulation in response to oscillations of a given peak frequency at a user defined phase shift from the detected signal. However, the UI is able to provide more conventional stimulation approaches in the form of square waves of a user defined intensity and duration. This section illustrates how to set up stimulation of this second type using the CANDO-CS UI, and refers to figure 4 to show visually where the option selections are on the UI.

1. Select the 'Manual' tab on the main UI (number 1 in figure 4)
2. Using the Stimulation type switch (number 2 figure 4) you can choose to either apply one of the closed loop algorithms in an open loop fashion, or to apply a direct value.
3. If you select 'Algorithm' in the Stimulation type switch, you can then manually control the on/ off times of the current closed loop algorithm using the Stimulation ON button (number 4, figure 4).
4. If you select 'Direct value' in the Stimulation type switch, you can then create an custom algorithm by selecting a Direct stimulation level from the slider, number 3 figure 4.
5. Once you have selected a Direct stimulation level you can either manually control this with the Stimulation ON button (pressing once will turn the stimulation on at the selected output level) or using the quick config On/ Off times. (NB: these quick config options require an initial algorithm configuration to appear, so if they are not visible for you then switch back to the Auto tab and click the Configure button, then once you have set up algorithm options you can return to the Manual tab to set up the direct value stimulation again.)

2.5 Using a test signal from a local machine

In order to test the functionality of the user interface without a serial device, or to make predictions of algorithm effectiveness using a model, it is possible to read in a test signal and have this processed by the user interface. The steps to use this functionality are as follows:

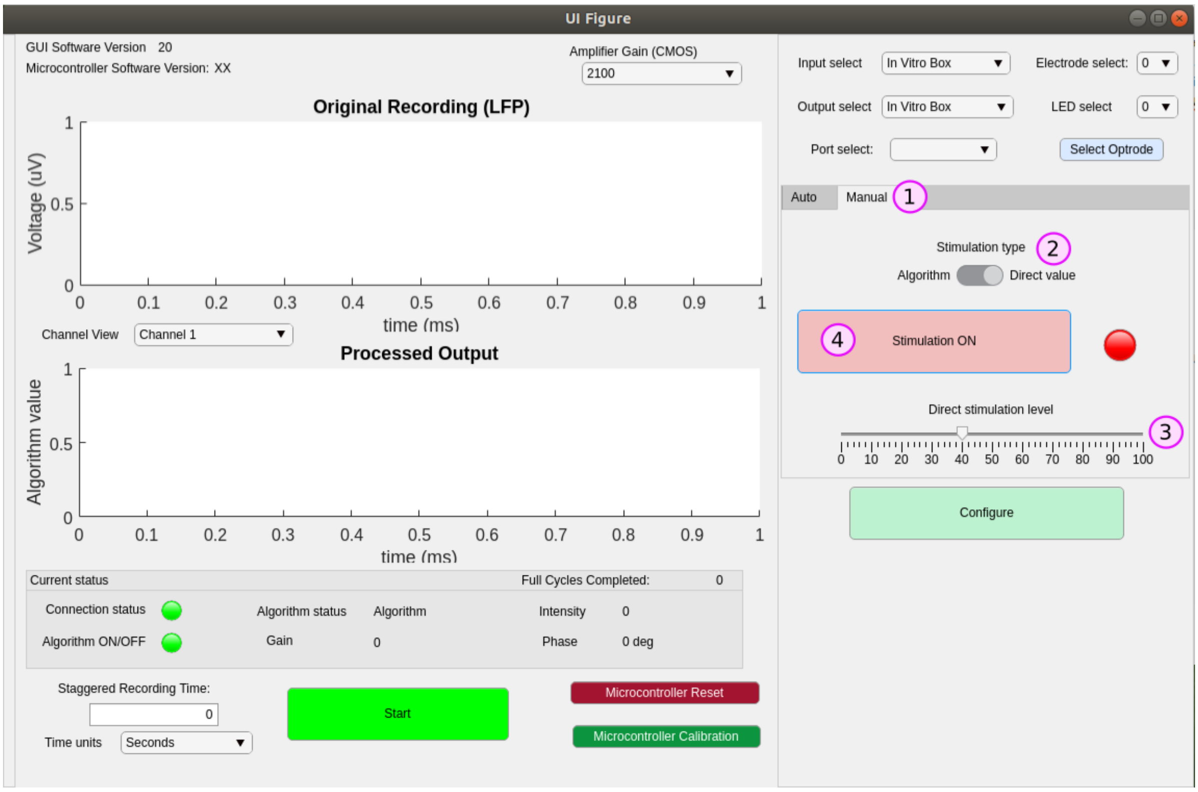

Figure 4: Screen Capture of the main GUI showing the manual stimulation options for setting up custom stimulation approaches, with numeric labels corresponding to information in the main text.

1. In a Matlab window, open and run the script 'signalGen' in the folder CAN-DOCS/GUI_Sources/Development_Archive/CompOnlyCode. This script will generate a signal outputted to a binary file called 'incoming.bin' in the current folder. Leave this running until you are finished with your test run. This script can be adapted to produce different signal types, by default the script creates a Wilson Cowan type Neural Mass Model replicating a signal recorded from a cortical column *in vitro*.

2. From the Input select drop-down menu (number 2 figure 2) select the option Test Signal

3. A pop up file selection window should appear, allowing you to select the binary output file generated by the test signal generator script ('incoming.bin' by default).

4. From the Output select menu (number 3 figure 2) select Static File Output, and

you will be prompted to select or create an output file for the input data and processed algorithm output to be saved to for later analysis.

5. If you wish to observe what stimulation might look like, either use the Configure button to select a closed loop algorithm, or the Manual tab to create an open loop algorithm (as per the above section).

6. Press the Start button once you are ready to begin. After enough time has passed for the input buffer to fill (default is 8000 bits which is a seconds worth of data from the CANDOCS microcontroller) the signal should begin to appear on the Original Recording graph (number 6 figure 2). If you have set up stimulation then you should also see an algorithm output applied in the Processed Output graph (number 7 figure 2). The algorithm, input and metadata will be saved in the file you selected or created during output selection.

3 Extending the CANDO-CS

3.1 Extending the user interface

The current application has been implemented in Matlab, as it has inbuilt functions for interfacing with serial devices and can be compiled to an application. We use Matlab's App Designer which provides a drag and drop interface for adding components. There is a code view which allows a developer to add callback functionality for any components, as well as custom functions. The Experimental Planner is a separate app that is called from the main app interface.

In order to add new functionality to the user interface, the App Designer drag and drop interface will allow new buttons/ text fields/ labels etc. to be easily added and then functionality for these can be added in the code view following existing examples.

In order to add parameter options for a new algorithm, we would recommend extending the Experimental Planner app, including additional algorithms in the algorithm select drop-down (number 4 figure 3) and then adding a corresponding parameter panel to appear on the selection of the new algorithm. This will need to correspond with changes in the firmware to respond to the new algorithm parameters. Currently, the firmware expects a parameter packet of 35 bytes, including a trigger flag in the final byte to tell the firmware how to respond to the current parameter packet. If your new algorithm uses alternative parameters to the existing ones, then these can be passed to the microcontroller by adding if statement catches for your new algorithm to the 'passParameters' function and the 'updateParameters' function in the main $CANDO\_control\_GUI$ app. See the section on adding new algorithms to firmware in order to make corresponding changes in the microcontroller.

3.2 Adding new algorithms to firmware

The firmware is written in embedded C, and includes modular functions for the existing algorithms. If you wish to add a new algorithm then adding a new function in the form of the existing functions is recommended. The firmware expects a 35 byte package from the UI, with the final bit acting as a trigger value to be read first and determine how the firmware reacts to the incoming packet. If you wish to add alternative algorithms then in addition to adding a function for the algorithm you will need to create a new trigger value as well as an if statement to detect the trigger and cause your custom algorithm to be processed.

4 Additional Features

1. Microcontroller reset
   This button will send a reset signal to the Microcontroller. If there is a bit error in the signal being sent across then the displayed LFP and algorithm will start to look strange, and resetting the microcontroller can resolve this issue.

2. Microcontroller Calibration
   This button will prompt a user to select a calibration file which will give a set of LED output levels to the microcontroller for testing a new output source (e.g. a new optrode) with a standard output level from the microcontroller. We provide a sample calibration file in the github repository.

3. Channel View selection
   If you have multiple inputs to the CANDOCS, the box will select one channel to display and to use to process closed loop algorithms. This can be changed with the channel view selection and the Electrode Select drop downs. The Channel View selection will change the electrode channel that is displaying on the plot (number 6 figure 2) but will not change the channel used for the algorithm. It provides a user the opportunity to determine which channel is more suitable to use before selecting a channel to use for the algorithm.

4. Electrode Select
   If you have multiple inputs then you can select which input channel to use for the algorithm using the Electrode Select drop-down menu. See above for how to view different channels in order to make an informed selection.

5. Amplifier Gain (CMOS)
   The CANDO custom CMOS has a variable amplifier gain that can be set using this drop-down box. If using custom hardware of your own then the options for this amplifier can be changed in the Matlab source code to adapt this for other custom devices using the 'AmplifierGainCMOSDropDownValueChanged' callback.

6. LED Select
   If you are using a device with multiple outputs (e.g. multiple LEDs on an optrode

shank as with the custom CANDO optrode devices, rather than a single commercial fibre optic device) then this drop down menu enables you to select a single LED output, or to have the algorithm output sent to all LEDs simultaneously. For a more fine grained selection see ‘Select Optrode’ below.

7. Select Optrode
   This option was designed for the CANDO custom optrodes, however it can be extended for alternative output devices. When this button is pressed a new UI window will appear with an optrode schematic (see figure **??**). The Select Optrode drop-down provides a number of preset options, and custom outputs can be added here. The selected device schematic is shown with check boxes over LED locations so that any combination of outputs can be selected. Electrode locations are also shown, and a single electrode can be selected as the Algorithm Electrode using the radio buttons on the left side panel.

5 Analysing results

The UI will save data in a series of *.mat* files. We have provided Matlab scripts in the github for combining these initial output files into results structures that can then be more easily plotted and analysed.

1. Initial Processing
   The function ‘run script.m’ will take a folder with these outputted .mat files and convert them into a results structure saved into a *.mat* file.

2. Filter and plot results
   The saved results structure can then be loaded, and fed into the function ‘filtAndPlot’ along with the sampling frequency (1000Hz by default). This function will bandpass filter the signal to remove noise above 40Hz and below 4Hz. NB: This box was developed in the UK at Newcastle University, so we filter at this level to ensure 50Hz AC noise is removed. If you are in a country where the AC mains frequency is 60Hz you may wish to adjust this level. The algorithm output, phase conditions and filtered signal are then plotted to give an overview of the data collected.

3. Frequency analysis
   We additionally supply frequency analysis scripts using the pwelch function to show the power/frequency graph for different phase conditions, along with the power modulation compared to the no-stimulation condition. See the function ‘frequency_analysis_cando’. This function is designed for experiments using the inbuilt closed loop algorithms, and code would need adapting for alternative experiments, however we hope that this will provide users with a good starting point for their own analysis.

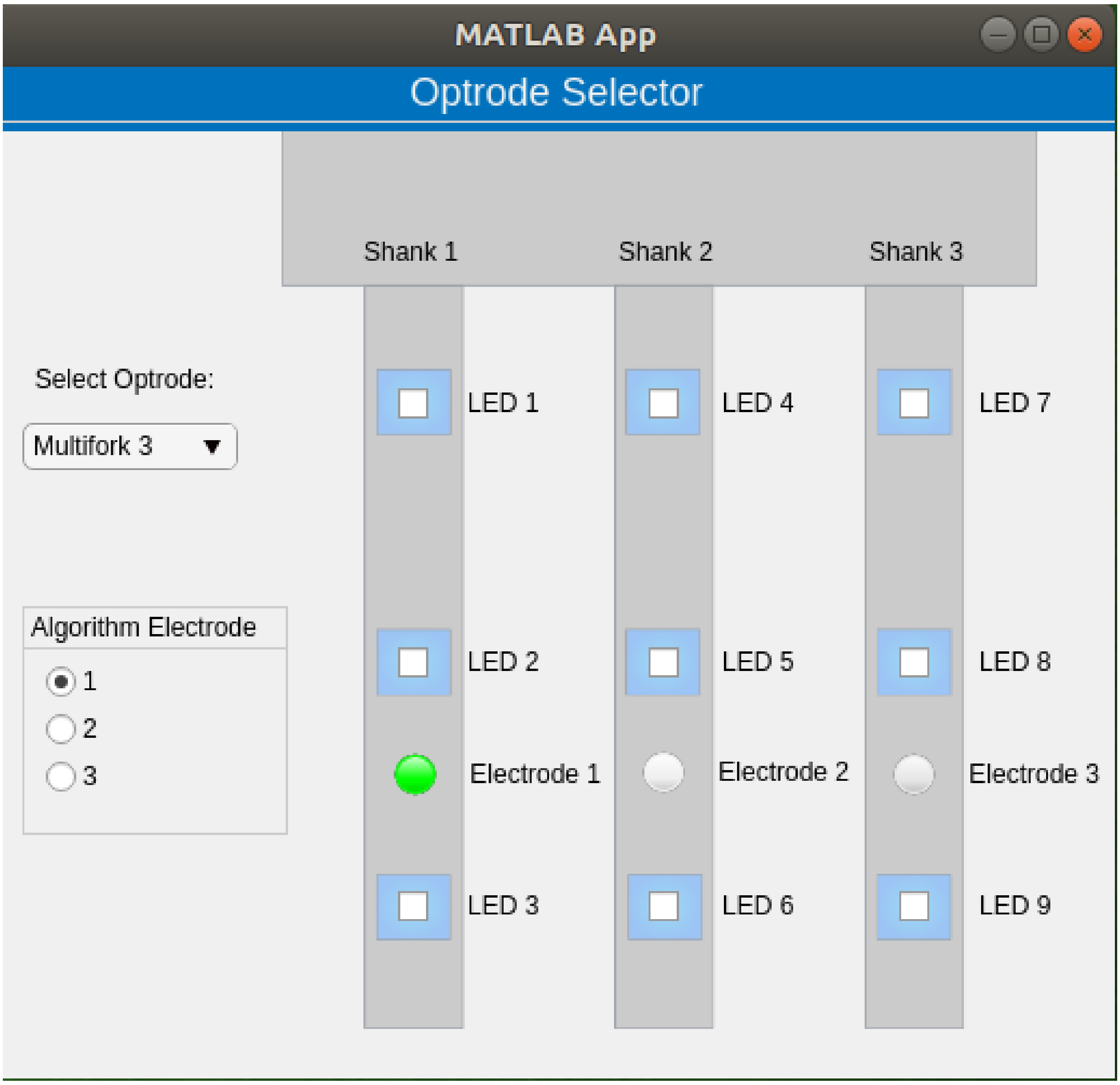

Figure 5: Screen Capture of the Optrode Selector GUI showing the interactable LED and electrode selector for an example optrode device.

4. Example analysis script
   We provide an example script for some data recorded using the CANDOCS in the folder CANDOCS/Analysis, 'CANDOCS_Example_Analysis' which runs through the above functions with some sample data generated in an experiment following the pattern of section 2.2 'Example experimental setup'.
