# Supplementary material for: A Closed-Loop Optogenetic Platform
Source: Front Neurosci. 2021 Sep 10;15:718311. doi: 10.3389/fnins.2021.718311 (PMC8462298; doi:10.3389/fnins.2021.718311)
Supplement: Supplementary file 2 [file Data_Sheet_2.PDF]

Supplementary Material

1 SUPPLEMENTARY FIGURES

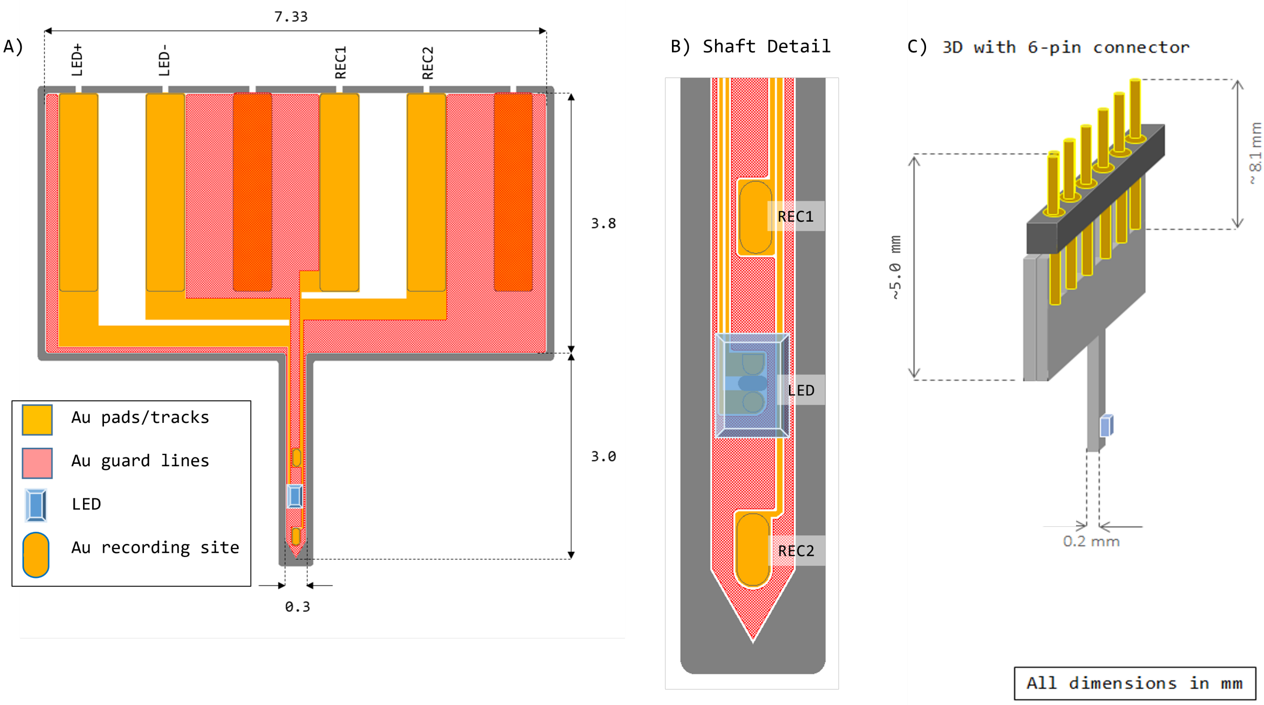

Figure S1. A) Layout and dimensions of the optrode indicating the Au pads/tracks (yellow), Au guard lines (pink), LEDs (blue) and Au recording sites (amber); B) Close-up of the shaft layout indicating the locations of the LED, recording sites, tracks and guard lines; C) 3D representation of the optrode with a 6-pin connector, including the physical dimension.

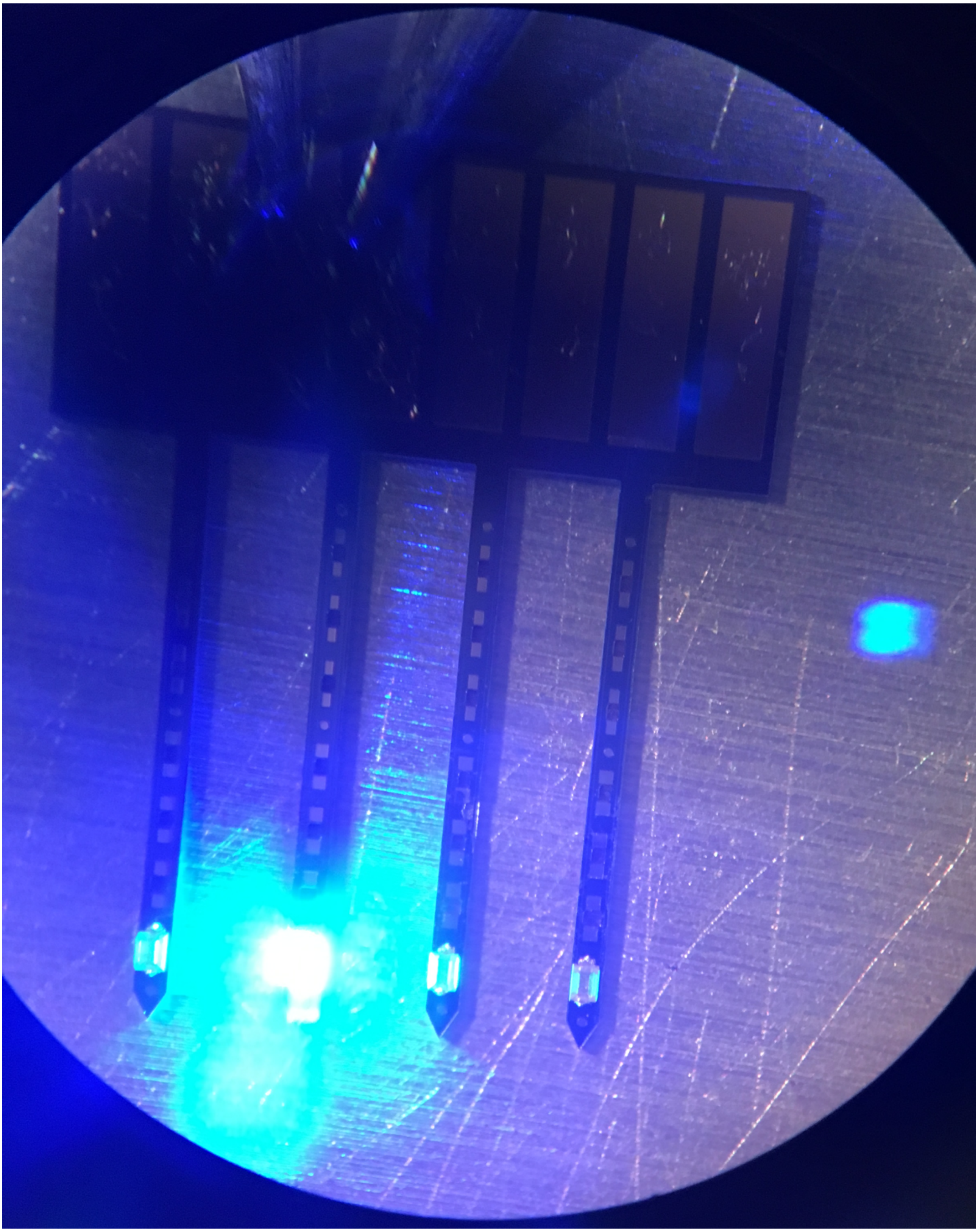

Figure S2. A fork with four shanks 5.0 mm long and 0.3 mm wide containing LEDs and Au recording sites. See Figure S1A and S1B for manufacturing details, as the fork follows a similar process.

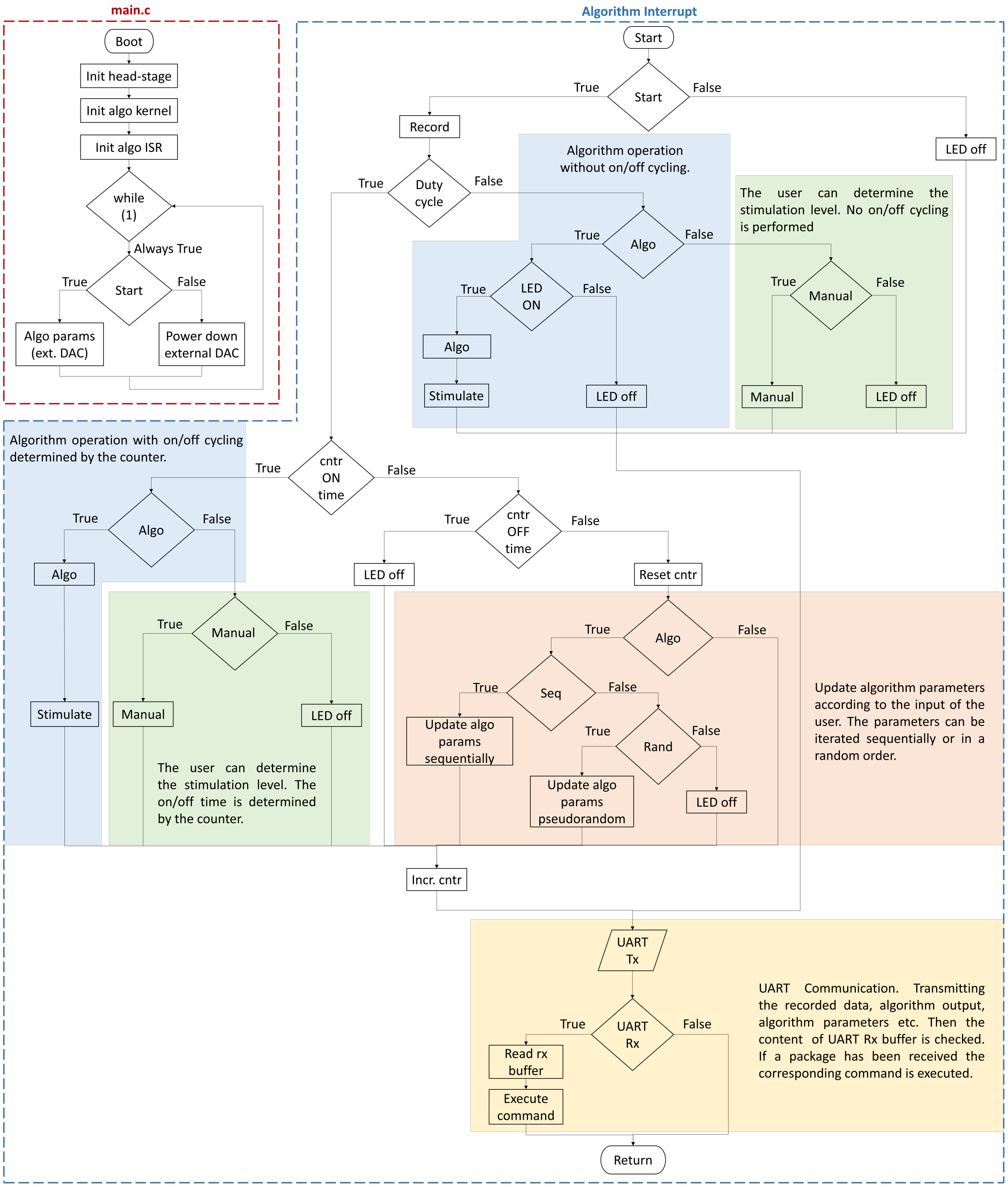

Figure S3. Detailed flowchart of the execution of the main.c (red outline) and Algorithm Interrupt Service Routine (blue outline). The blue backgrounds indicate parts of the code where the algorithm operation is determined with and without an on/off period. Similarly, the green backgrounds indicate the parts of the code where the stimulation level can be manually determined by the user with or without an on/off period. The light red background refers to the part of the code where the update of the parameters of the algorithm is performed; this can be done sequentially or randomly. Finally, the yellow background indicates the part of the code responsible for the Universal Asynchronous Receiver-Transmitter (UART) communication; both transmission and reception of data packets.

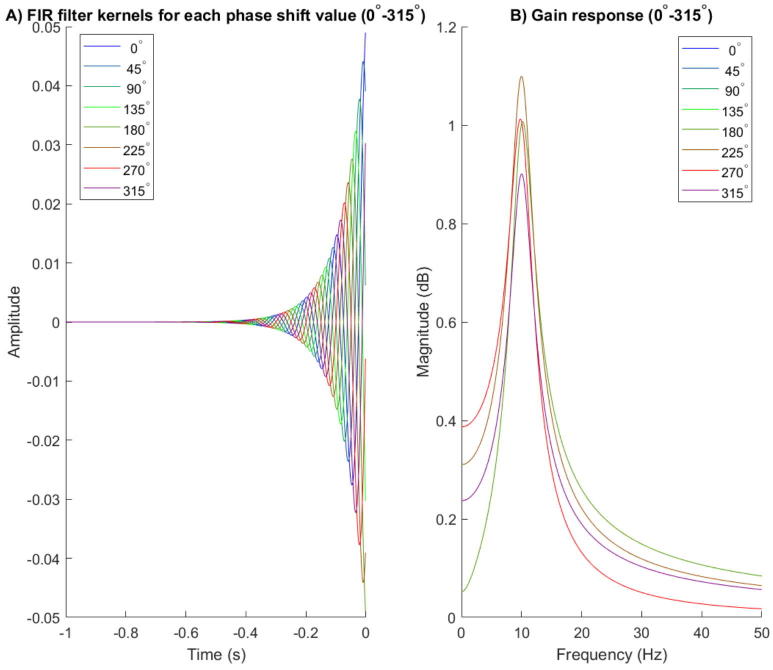

Figure S4. A) Exemplar FIR filter kernels for different phase-shifts, ranging between 0° to 315°, with a 45° step. Each kernel has a size of 512 taps; B) Kernel gain response for each phase (0°-315°) idicating a central frequency of 10 Hz; C) Phase response of the FIR filter kernels.

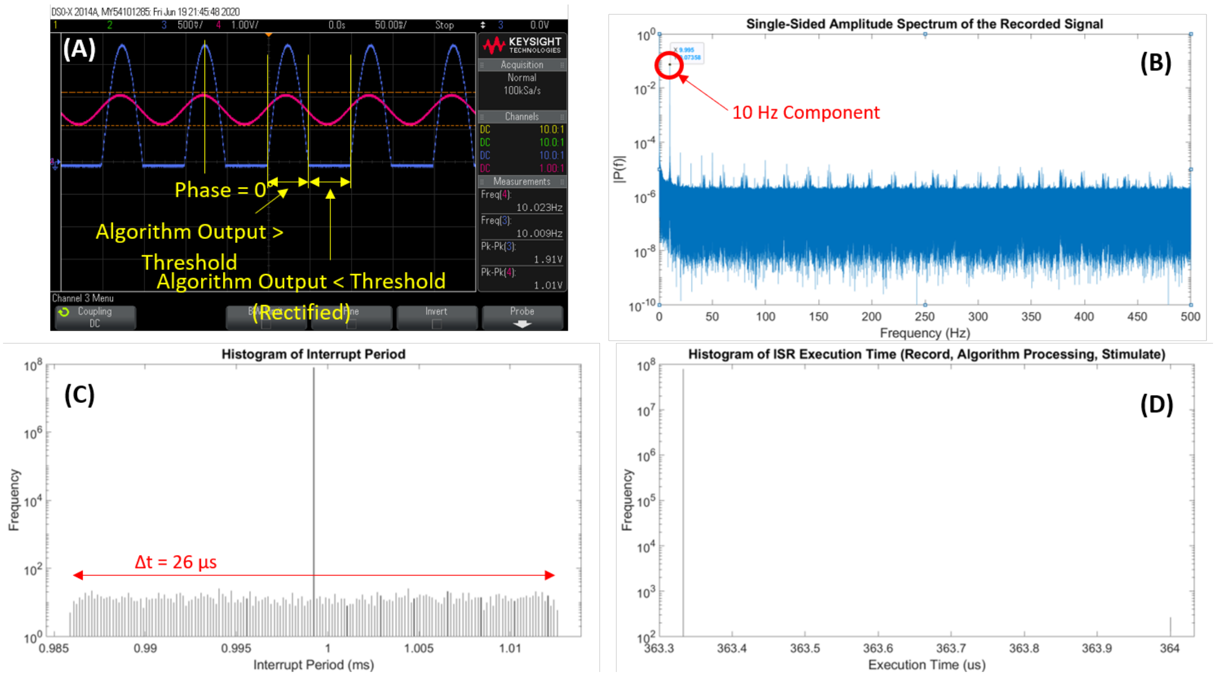

Figure S5. Stability testing performed over a 24h period, to ensure a robust and reliable operation of the CANDOCS. A) Input (pink) 1V_{pp}, 10 Hz sine wave signal and algorithm output (blue), which is a rectified and phase shifted (0° phase shift in this case) version of the input signal; B) Power spectrum of the recorded signal indicating that the main power component was 10 Hz; C) Histogram of the duration of the interrupt period, centered around 0.998 ms and deviating by +/- 13 μs; D) Histogram of the execution time of the Interrupt Service Routine (ISR).
